# Absence of nuclear receptors LXRs impairs immune response to androgen deprivation and leads to prostate neoplasia

**DOI:** 10.1371/journal.pbio.3000948

**Published:** 2020-12-07

**Authors:** Laura Bousset, Amandine Septier, Julio Bunay, Allison Voisin, Rachel Guiton, Christelle Damon-Soubeyrant, Yoan Renaud, Angélique De Haze, Vincent Sapin, Anne Fogli, Amandine Rambur, Cyrille De Joussineau, Ayhan Kocer, Amalia Trousson, Joëlle Henry-Berger, Marcus Höring, Gerhard Liebisch, Silke Matysik, Jean-Marc A. Lobaccaro, Laurent Morel, Silvère Baron

**Affiliations:** 1 Université Clermont Auvergne, GReD, CNRS UMR 6293, INSERM U1103, Clermont-Ferrand, France; 2 Centre de Recherche en Nutrition Humaine d’Auvergne, Clermont-Ferrand, France; 3 Institute of Clinical Chemistry and Laboratory Medicine, Regensburg University Hospital, Regensburg, Germany; Institute of Cancer Research, Chester Beatty Laboratories, London, UNITED KINGDOM

## Abstract

Chronic inflammation is now a well-known precursor for cancer development. Infectious prostatitis are the most common causes of prostate inflammation, but emerging evidence points the role of metabolic disorders as a potential source of cancer-related inflammation. Although the widely used treatment for prostate cancer based on androgen deprivation therapy (ADT) effectively decreases tumor size, it also causes profound alterations in immune tumor microenvironment within the prostate. Here, we demonstrate that prostates of a mouse model invalidated for nuclear receptors liver X receptors (LXRs), crucial lipid metabolism and inflammation integrators, respond in an unexpected way to androgen deprivation. Indeed, we observed profound alterations in immune cells composition, which was associated with chronic inflammation of the prostate. This was explained by the recruitment of phagocytosis-deficient macrophages leading to aberrant hyporesponse to castration. This phenotypic alteration was sufficient to allow prostatic neoplasia. Altogether, these data suggest that ADT and inflammation resulting from metabolic alterations interact to promote aberrant proliferation of epithelial prostate cells and development of neoplasia. This raises the question of the benefit of ADT for patients with metabolic disorders.

## Abstract 2

Mice lacking the liver X nuclear receptors (LXRs), crucial integrators of lipid metabolism, were used to study the response of the prostate to androgen deprivation. This reveals that lack of androgens leads to chronic inflammation due to impaired clearance of castration-induced apoptotic cells, allowing production of pro-inflammatory cytokines and promoting prostate neoplasia.

## Introduction

It is becoming increasingly clear that inflammation contributes to prostate cancer. This is supported by studies of genetically engineered mouse models, which demonstrate that chronic inflammation of the prostate can lead to development of neoplasia [1,2]. In most cases, prostate inflammation can be the result of bacterial or viral infections [3]. However, emerging evidence suggests that obesity as well as metabolic syndrome are also associated with systemic inflammation, a concept called metaflammation [4]. Consistent with this idea, mice fed a high-fat diet present systemic low-grade inflammation, and nuclear factor kappa B (NF-kB)-dependent chronic prostate inflammation [5]. In human, chronic inflammation of the prostate is characterized by infiltration of mostly CD4^+^ T cells (70% to 80%), B cells (10% to 15%), and macrophages [6]. A review of the literature suggests an association between an inflammatory state and prostate cancer development in patients, but there is still no evidence of causal relationship [7]. In support of this idea, a meta-analysis found a correlation between the presence of metabolic syndrome and a worse outcome of prostate cancer, but only observed a weak association with prostate cancer incidence [8]. Consistent with this, a recent study in metastatic Castration-Resistant Prostate Cancer (mCRPC) patients, identified a high-risk prognostic group based on criteria of metabolic syndrome and inflammation, which was associated with decreased progression-free and overall survival [9]. This is further supported by a prospective study that demonstrated an increased risk of high-grade and advanced prostate cancer in patients with metabolic syndrome [10]. Altogether, these data suggest that obesity and metabolic syndrome could be associated with cancer initiation and progression, through stimulation of inflammation. However, the links between lipid metabolism, cytokines, and inflammation-related factors released by immune cells and tumorigenesis within the prostate are poorly understood.

Nuclear receptors liver X receptors (LXRs) are integrators of lipid metabolism and inflammation response [11]. They were first described for their role in pathophysiology of atherosclerosis as endogenous inhibitors of atherosclerosis by limiting lipid overload in macrophages [12]. Effectively, these transcription factors control expression of cholesterol and lipid metabolism related genes, such as cholesterol efflux cassette transporters ABCA1 and ABCG1/5/8, fatty acid synthase (FASN), or apolipoprotein (APOE). Most importantly, LXRs also negatively regulate inflammatory response by down-regulating pro-inflammatory cytokines coding genes expression, especially in macrophages [13–15]. However, reciprocal interactions of lipid metabolism and inflammation mediated by LXRs has not been extensively analyzed in the context of prostate cancer.

Classically, prostate cancer is treated by androgen deprivation therapy (ADT) to induce tumor regression. If it is now well known that ADT induces apoptosis of prostate tumor cells and adjacent normal cells, thus allowing tumor regression, ADT can also affect tumor immune microenvironment. Many studies show an increasing infiltration of immune cells in prostate tumors in response to androgen deprivation [16–18]. Moreover, an elevated infiltration of macrophages after ADT is associated with an increased risk of biochemical recurrence [17,18]. However, the relationship between ADT-induced immune cells infiltration, prostate inflammation, and cancer initiation has not been evaluated. More generally, this raises the question of an aberrant response of the immune microenvironment to ADT, in the context of metabolic alteration and its potential role as a paracrine stimulator of epithelial tumorigenesis and tumor recurrence.

In the present study, we investigated prostate response to androgen deprivation in a mouse model invalidated for LXRs. We demonstrate that castration induces chronic inflammation of LXRs-null prostates. This is associated with recruitment of macrophages defective for apoptotic cells clearance, which in turn allows production of inflammatory cytokines. Among them, we show that osteopontin (OPN) induces proliferation of epithelial cells in a paracrine manner. As a result, chronic inflammation observed in LXRs-null prostates stimulates the proliferation rate of epithelial cells, which progressively form neoplastic precancerous lesions.

## Results

### Invalidation of nuclear receptors LXRs impairs regression of prostate in response to androgen deprivation

**Fig 1:**
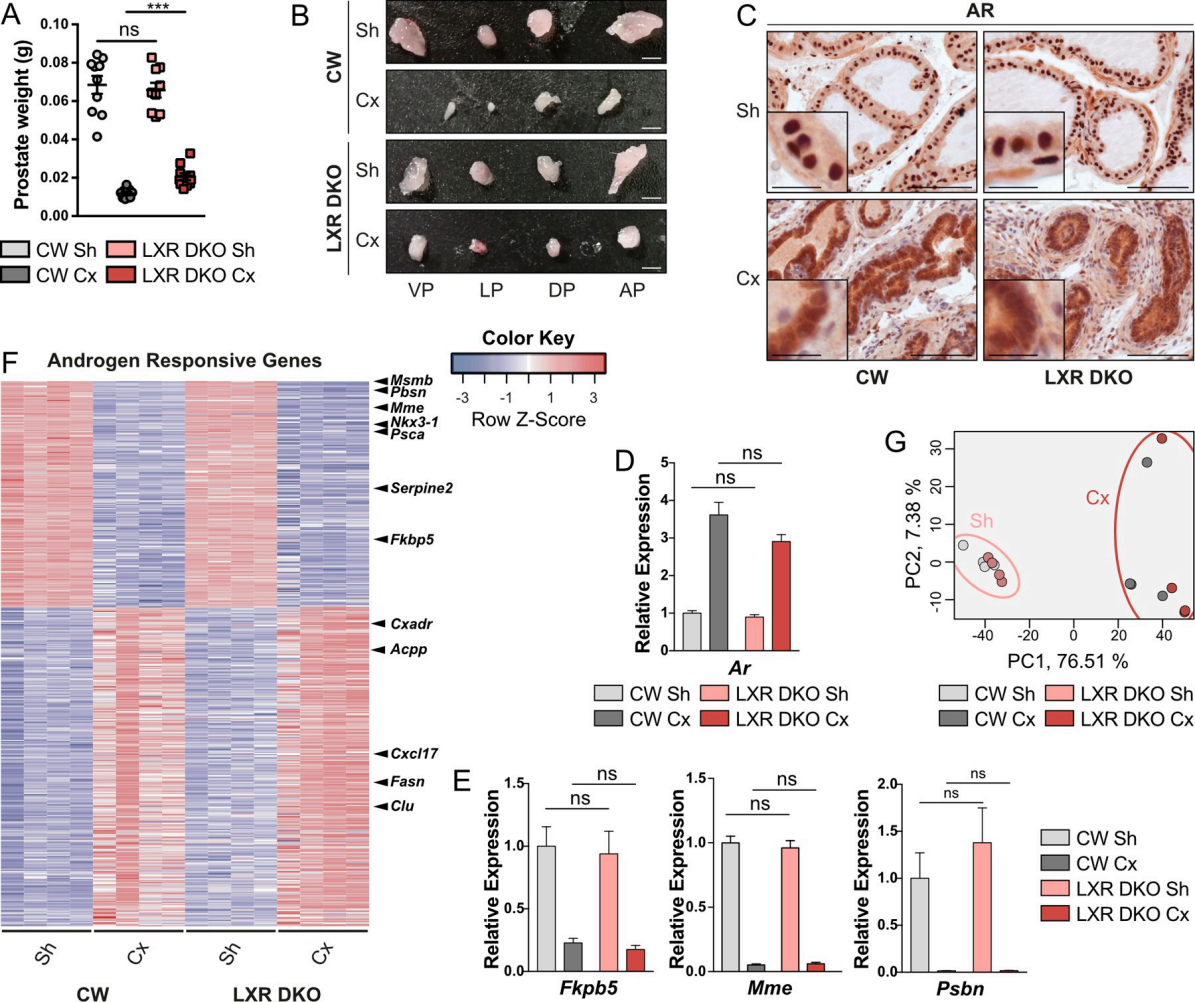
LXR deficiency impairs response to androgen deprivation in an androgen-independent manner. (A and B) Whole prostate weight analysis and (B) macroscopic observation of VP, LP, DP, and AP prostate lobes of 1-month Cx or Sh male CW and LXR DKO mice reveal a marked difference in response to castration in the absence of LXRs. (C) Immunohistochemical detection of AR showing nuclear to cytoplasmic relocalization following 1-month castration. (D and E) RT-qPCR analysis of mRNAs encoding *Ar* (D) and its target genes *Fkbp5*, *Mme*, and *Pbsn* (E). (F) RNA sequencing analysis of ARG demonstrates no difference in gene expression changes in response to 1-month castration between LXR DKO and CW prostates. (G) Principal component analysis based on ARG expression confirms distinct clustering of Cx mice from Sh mice. Groups are composed of at least 4 animals. Bars represent mean ± SEM. Statistical analyses were performed via Mann–Whitney test. *p < 0.05, **p < 0.01, ***p < 0.001 and ns. Scale bars, 100 μm; insets, 20 μm. For numerical raw data, please see S1 Data. For supporting dataset, please see S2 Data. AP, anterior; ARG, androgen responsive genes; Cx, castrated; CW, control wild-type; DP, dorsal; LP, lateral; LXR, liver X receptor; LXR DKO, LXR alpha and beta double knock-out; ns, nonsignificant; RT-qPCR, quantitative reverse transcription PCR; Sh, sham-operated; VP, ventral.

To evaluate how LXRs invalidation can affect response to androgen deprivation, we performed 1-month castration on LXRs-null mice in comparison with LXRs-sufficient mice. Consistent with previously published data [19], we found no difference of whole prostate weight and macroscopic aspect between LXR-sufficient (noted as CW) and LXR-null groups (noted as LXR DKO), in the absence of metabolic stress (Fig 1A and 1B). As expected, we observed a drastic reduction in whole prostate weight of CW mice in response to 1-month castration. Regression of the highly androgen-sensitive seminal vesicles further confirmed efficacy of castration (Figs 1A and 1B and S1). Interestingly though, LXR DKO prostates were 2-fold heavier than CW, following 1-month castration (Fig 1A and 1B). This suggested hyposensitivity of LXR knock-out mice to androgen deprivation. To identify the underpinnings of this hyporesponse to castration, androgen receptor (AR) expression was analyzed by immunohistological detection. AR was expressed in epithelial cells as well as some stromal cells and presented the expected relocalization from nuclei to cytoplasm in the absence of androgens, independent of LXRs status (Fig 1C). Likewise, *Ar* mRNA accumulation was increased in response to castration but did not differ between LXR DKO and CW prostates in castrated or sham-operated conditions (Fig 1D). Expression of direct AR target genes *Fkbp5*, *Mme*, and *Pbsn* was strongly down-regulated in response to castration both in CW and LXR DKO prostate (Fig 1E). However, despite this strong effect of castration, AR target genes exhibited a similar expression pattern whatever the genotype. This suggested that aberrant response to castration in LXR DKO prostates was not associated with aberrant AR signaling. To further evaluate this hypothesis, we performed RNA sequencing analysis of the 4 different models and identified an androgen-responsive genes signature (ARG signature) as genes differentially expressed in response to androgen deprivation in CW prostates (S2 and S3 Datas). Interestingly, modulation of expression of the ARG signature was equivalent in CW and LXR DKO prostates in response to 1-month castration (Fig 1E). Principal component analysis relying on the ARG signature confirmed that castrated mice clustered separately from sham-operated mice, independently of LXRs status (Fig 1F). Therefore, we concluded that the milder regression in LXR DKO–castrated mice occurred independently of the androgen signaling pathway.

### Androgen deprivation induces immune cells infiltration mostly composed of F4/80^+^ macrophages

**Fig 2:**
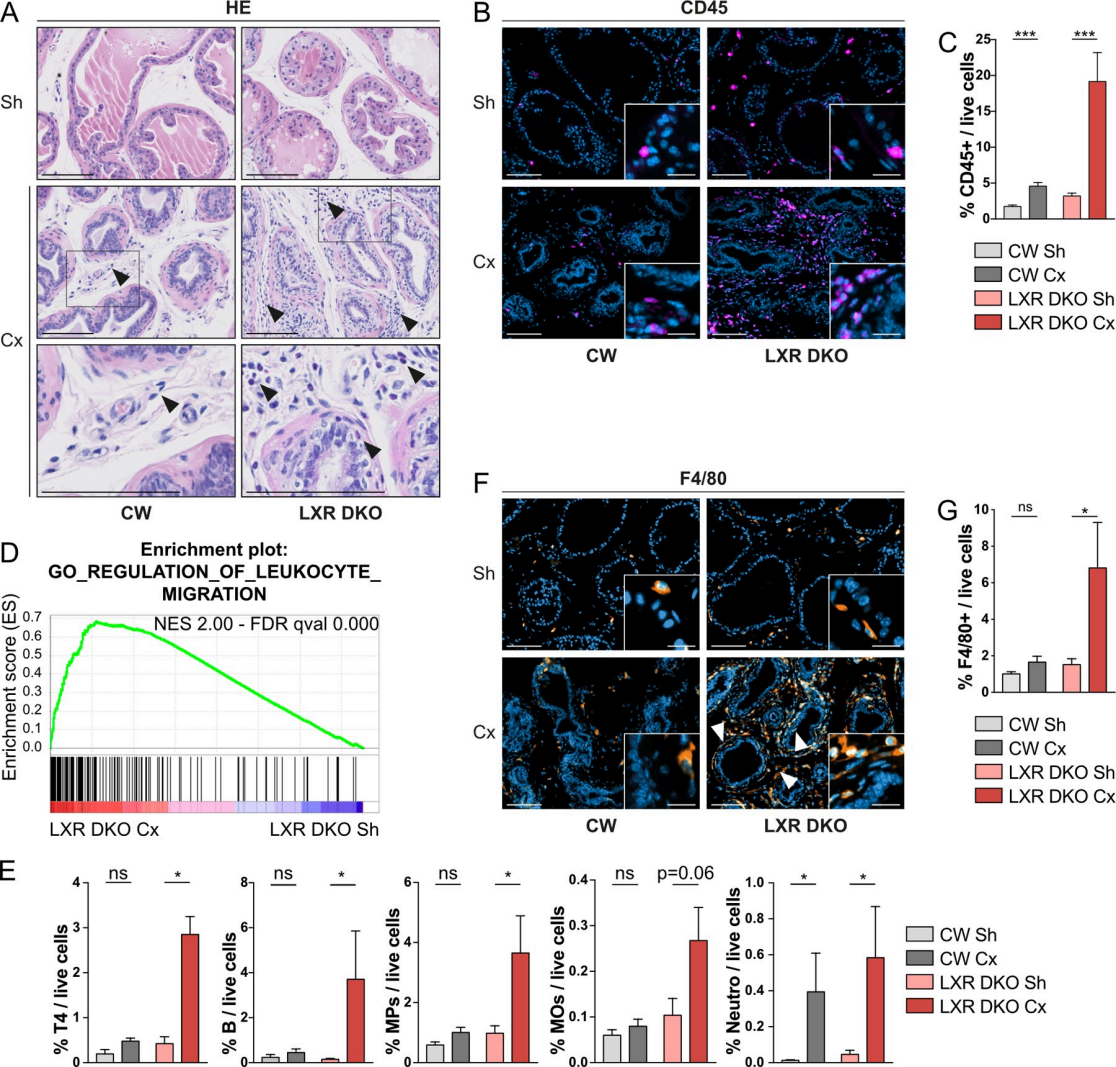
Androgen deprivation-induced immune infiltration in LXR-null prostates is composed of a majority of F4/80^+^ macrophages. (A) Hematoxylin and eosin staining of prostates from 1-month Cx or Sh male CW and LXR DKO mice. Arrowheads indicate immune cells infiltration. (B) Immunohistological staining of the pan-leucocytes marker CD45 in 1-month Cx or Sh CW and LXR DKO prostates. (C) Flow cytometry analysis of CD45+ leucocytes representation in whole prostates. (D) Gene ontology analysis of RNA sequencing reveals enrichment in regulation of leukocyte migration gene set, after 1 month of castration in LXR DKO mice. (E) Castration of LXR DKO mice induces a marked increase in infiltration of T4 lymphocytes, B cells, MPs, and MOs in comparison to CW mice. CD45^+^ immune cells were defined as: CD4+ T4 lymphocytes, CD19+ B cells, CD11b^+^ Ly6C^−^ Ly6G^−^ F4/80^+^ SCC^low^ MOs, CD11b^+^ Ly6C^−^ Ly6G^−^ F4/80^−^ and CD11b^−^ CD11c^−^ F4/80^+^ other MPs, and CD11b^+^ Ly6C^−^ Ly6G^+^ neutro. (F and G) Immunohistological staining (F) and flow cytometry analysis (G) of F4/80^+^ MOs representation in 1-month Cx or Sh CW and LXR DKO prostates. Groups are composed of at least 4 animals. Bars represent mean ± SEM. Statistical analyses were performed via Mann–Whitney test. *p < 0.05, **p < 0.01, ***p < 0.001 and ns. Scale bars, 100 μm; insets, 20 μm. For numerical raw data, please see S1 Data. For supporting dataset, please see S2–S4 Datas. For flow cytometry raw data, please see S1 FlowCytometry RawDataFCS. Cx, castrated; CW, control wild-type; LXR, liver X receptor; LXR DKO, LXR alpha and beta double knock-out; MOs, macrophages; MPs, mononuclear phagocytes; neutro, neutrophils; ns, nonsignificant; Sh, sham-operated.

To further understand hyporesponse to castration in LXR DKO mice, we performed hematoxylin eosin staining of tissue sections. Interestingly, we observed an increased number of mononuclear cells in the stromal compartment in response to castration, both in CW and LXR DKO prostates (Fig 2A). However, this was further increased in LXR DKO prostates and was accompanied by the presence of eosinophilic cells. This suggested immune cells infiltration. Consistent with this hypothesis, immunohistological detection of CD45 pan-leukocyte marker confirmed increased immune cells infiltration in response to castration in LXR DKO mice, compared with CW (Fig 2B). Estimation of immune cells infiltration within the prostates was further refined by flow cytometry. In these analyses, CD45^+^ leukocytes represented more than 19% of live cells in LXR DKO castrated prostates (6-fold increase compared to sham-operated LXR DKO), whereas this proportion was reduced to 5% in CW castrated prostates (2.6-fold increase compared to sham-operated CW) (Figs 2C and S2). These data showed that LXR DKO mice presented a massive increase in immune cells infiltration in response to androgen deprivation, which was associated with hyporesponse to castration compared to CW mice.

To gain insight into the relative contribution of LXR in prostate epithelium versus stroma, we developed a model of conditional LXRαβ ablation within prostate epithelial cells (*Lxrαβ* ^*pe-/-*^). Interestingly, these mice presented no signs of excessive immune cells infiltration in response to 1 month of castration (S3A Fig). Moreover, epithelial ablation of LXR did not induce obvious histological alterations or differential regression in response to castration (S2C and S3B Figs). This demonstrated that the differential response to castration observed in LXR DKO prostates compared to CW prostates was not the result of a cell-autonomous effect of LXR within epithelial cells, but rather involved LXR function in immune cells. To better characterize the immune infiltration in castrated LXR DKO prostates, we made use of RNA sequencing data. Gene Set Enrichment Analyses (GSEA) identified regulation of leukocyte migration as one of the most significantly deregulated gene sets in response to castration in LXR DKO prostates (Figs 2D and S4). To further characterize the immune infiltrate, we performed flow cytometry analyses on different immune populations. These showed that both lymphoid and myeloid cells were recruited in response to androgen deprivation, specifically in LXR-null mice (Fig 2E and S4 Data). Indeed, we found an increase in CD4^+^ T4 cells, in CD19^+^ B cells, and particularly in CD11b^+^ or F4/80^+^ mononuclear phagocytes and F4/80^+^/CD11b^+^ macrophages. This indicated that androgen deprivation had a prominent effect on phagocytes recruitment in the absence of LXRs. Besides, neutrophils recruitment in response to castration was equivalent in CW and LXR DKO prostates (Fig 2E). We further analyzed the localization of F4/80^+^ cells by immunohistochemistry. They were mostly present in the stromal compartment and fibromuscular layer surrounding acini even though some rare cells were found in epithelia (Fig 2F, arrowheads). Flow cytometry analysis confirmed that F4/80^+^ cells were specifically deregulated in LXR DKO castrated prostates to represent up to 7% of live cells within the tissue (Fig 2G). Altogether, these data indicated that androgen deprivation in LXR DKO mice resulted in a massive immune cells infiltration mostly composed of F4/80^+^ macrophages.

### Androgen deprivation promotes cholesterol accumulation in the prostate

**Fig 3:**
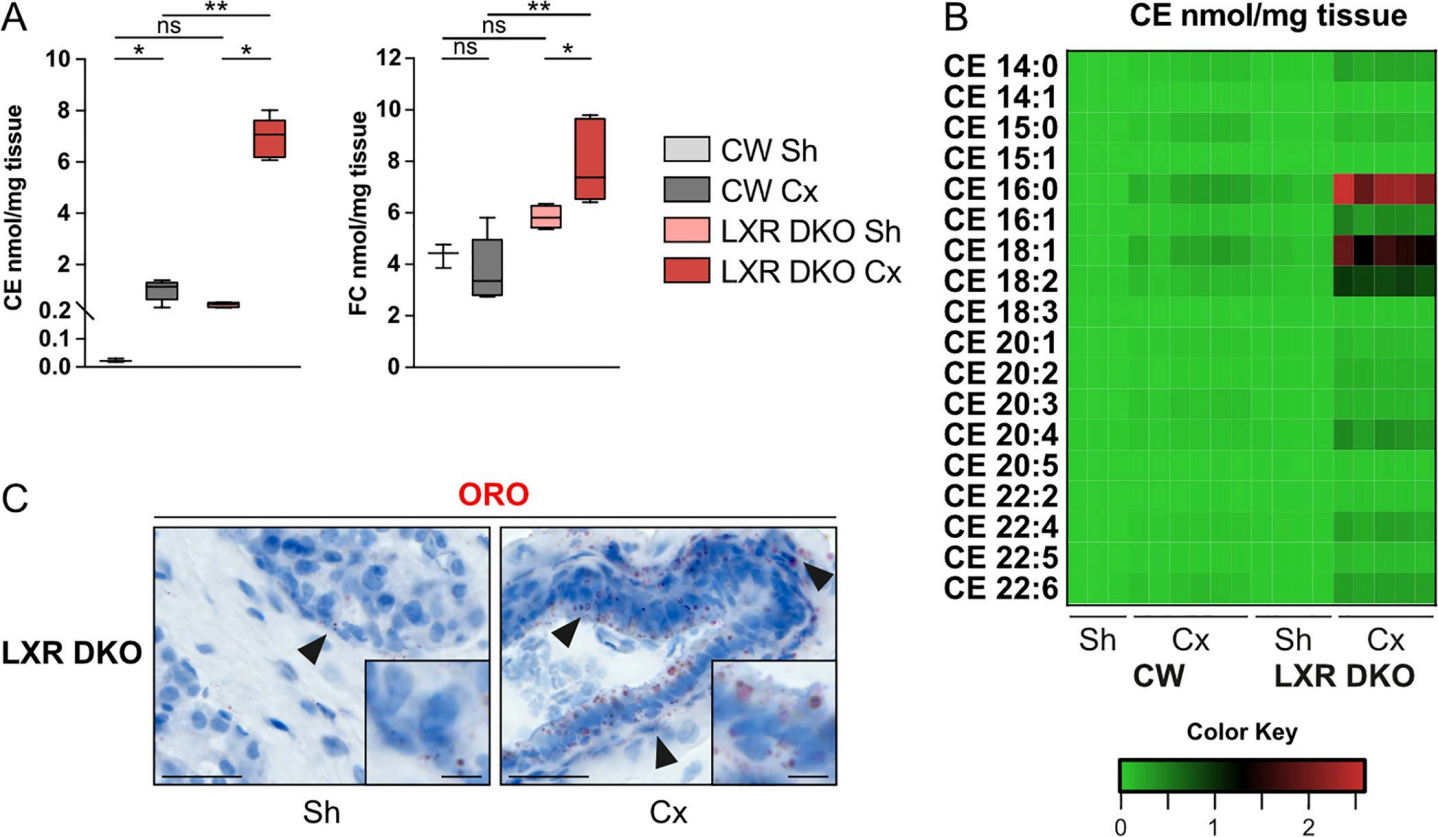
Cholesterol accumulation is associated with response to androgen deprivation in LXR DKO prostates. (A) Accumulation of CE and FC in the prostates of CW and LXR DKO mice following 1-month castration. (B) Heatmap showing accumulation of 18 species of CE in the prostates of CW and LXR DKO mice following 1-month castration. (C) Lipid accumulation analyzed by Oil-red-O staining in LXR DKO prostates following 1-month castration. For supporting dataset, please see S5 Data. CE, cholesteryl esters; CW, control wild-type; Cx, castrated; FC, free cholesterol; LXR DKO, LXR alpha and beta double knock-out; ns, nonsignificant; ORO, Oil Red O; Sh, sham-operated.

As LXRs nuclear receptors are important regulators of cholesterol homeostasis within the cell, particularly in macrophages, we then evaluated the cholesterol content of CW and LXR DKO prostates. Castration induced accumulation of cholesteryl esters (CE) in CW prostates (Fig 3A and S5 Data). Consistent with a role of LXR in regulation of cholesterol homeostasis in prostate [19], this phenomenon was dramatically increased in LXR DKO prostates (almost 8-fold compared to CW), which also accumulated free cholesterol following androgen deprivation (Fig 3A and S5 Data). Detailed analysis of CE species (Fig 3B and S5 Data) showed a dramatic increase in cholesteryl palmitate (CE16:0), oleate (CE18:1), and linoleate (CE18:2) in LXR DKO prostates following castration. Deregulation of cholesterol homeostasis was further confirmed by Oil-red-O staining showing a large accumulation of lipids both in the epithelium and stroma (Fig 3C).

Altogether, these data show that castration induces cholesterol accumulation in the prostate. This phenomenon is dramatically amplified by ablation of LXR and may be involved in immune infiltration.

### Castration-induced apoptotic prostate cells are not effectively eliminated in LXR-null mice

**Fig 4:**
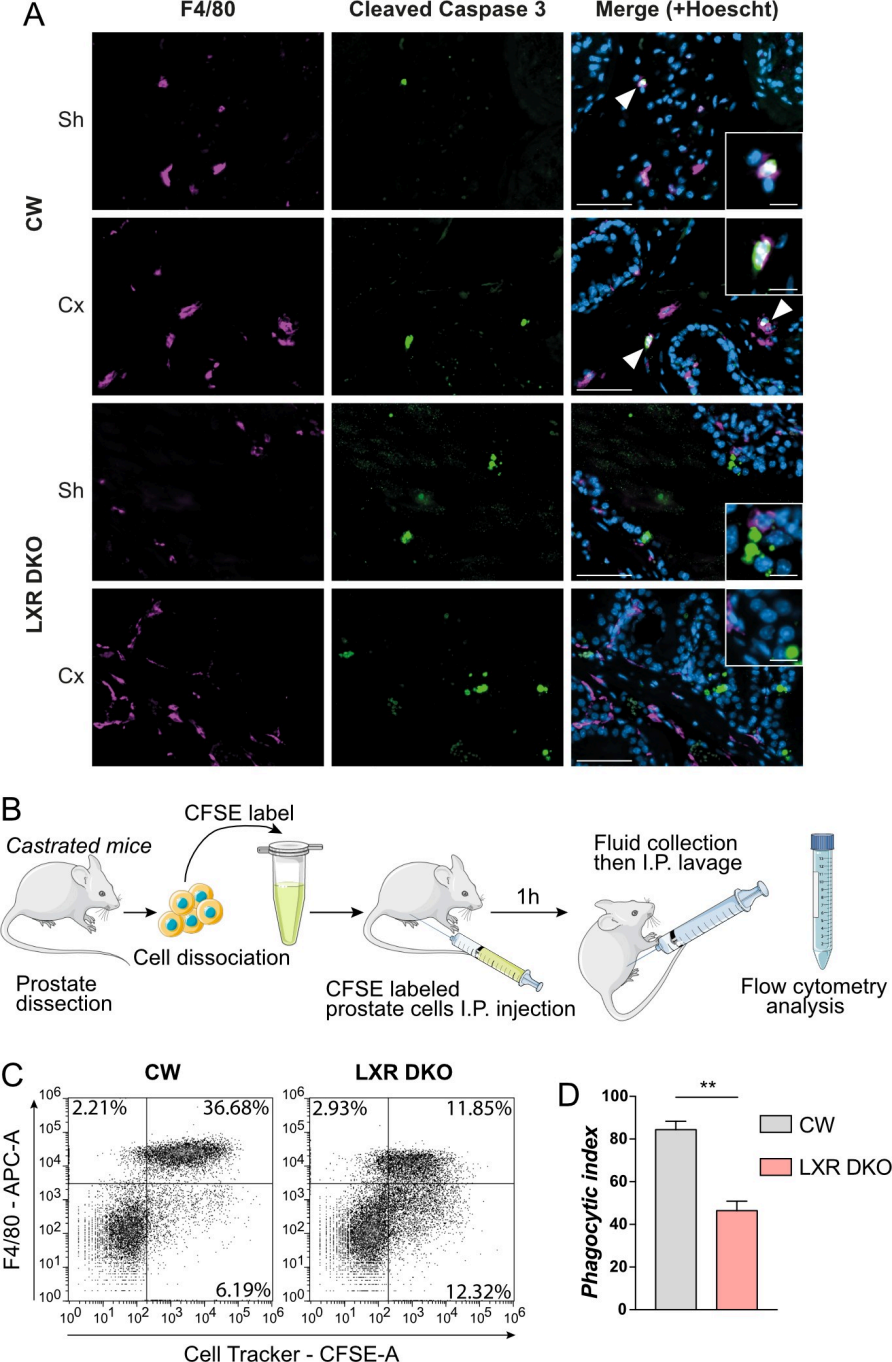
LXR-deficient mice present defective castration-induced apoptotic prostate cells clearance. (A) Immunohistological detection of macrophages marker F4/80^+^ and cleaved caspase 3-positive apoptotic cells in prostates of 1-week castrated mice or sham-operated mice indicates no colocalization of F4/80+ macrophages with cleaved caspase 3-positive apoptotic cells in LXR DKO mice in contrary to CW mice. (B) In vivo phagocytosis assay. CW or LXR DKO castrated for 4 days were culled, and whole prostates were dissected. Prostate cells were then dissociated and labeled with CFSE to allow tracking of cells from donor mice. Labeled cells were injected in the abdominal cavity of receiver mice and incubated for 1 hour. Abdomens of receiver mice were washed, and the collected fluid was analyzed by flow cytometry. (C and D) Flow cytometry analysis (C) of phagocytosed CFSE-labeled prostate cells by F4/80^+^ cells. Phagocytic index (D) shows a dramatic decrease of phagocytosis capacities of LXR DKO F4/80^+^ cells. Groups are composed of at least 4 animals. Bars represent mean ± SEM. Statistical analyses were performed via Mann–Whitney test. *p < 0.05, **p < 0.01, ***p < 0.001 and ns. Scale bars, 50 μm; insets, 10 μm. For numerical raw data, please see S1 Data. CFSE, carboxyfluorescein succinimidyl ester; CW, control wild-type; Cx, castrated; LXR, liver X receptor; LXR DKO, LXR alpha and beta double knock-out; ns, nonsignificant; Sh, sham-operated.

Macrophages play an important role in clearance of apoptotic cells. As androgen deprivation induces apoptosis of androgen-sensitive prostate cells [20,21], we then asked if LXR-deficient macrophages were able to eliminate these apoptotic cells. To evaluate the phagocytosis of apoptotic cells, we performed co-immunohistological detection of F4/80 and cleaved caspase 3 as a marker of apoptosis. In CW mice, apoptotic prostatic cells colocalized with F4/80^+^ macrophages after 1 week of castration, as well as in sham-operated mice (Fig 4A). This indicated that in basal condition, F4/80^+^ cells were efficient to clear apoptotic cells through phagocytosis. However, F4/80^+^ and cleaved caspase 3 double-positive cells were not observed in LXR DKO prostates after 1-week castration or sham operation (Fig 4A). This suggested that LXR-deficient macrophages were not recruited to apoptotic cells and may have impaired phagocytosis abilities. To evaluate this hypothesis, we performed an in vivo phagocytosis assay as described in Fig 4B. Prostates from CW or LXR DKO mice were dissected, and cells were dissociated before labeling with carboxyfluorescein succinimidyl ester (CFSE) cell tracker. Labeled cells from CW or LXR DKO mice were injected in the peritoneal cavity of mice of the corresponding genotype and incubated for 1 hour (Fig 4B). Peritoneal fluid was then collected, and macrophages were immunodetected with an antigen-presenting cell (APC)-labeled F4/80 antibody, before flow cytometry analysis. Most of cell tracker–labeled prostate cells were phagocytized by F4/80^+^ cells in control wild-type mice, whereas half of them were not in LXR DKO mice (Fig 4C). This was reflected by the phagocytic index, which was dramatically decreased when LXRs were invalidated (Figs 4D and S5). Altogether, these data indicate that F4/80^+^ macrophages are involved in clearance of castration-induced apoptotic prostate cells, a process that seems impaired in LXR-deficient mice.

### Phagocytosis-deficient macrophages infiltration is associated with chronic inflammation of LXR-null prostate

**Fig 5:**
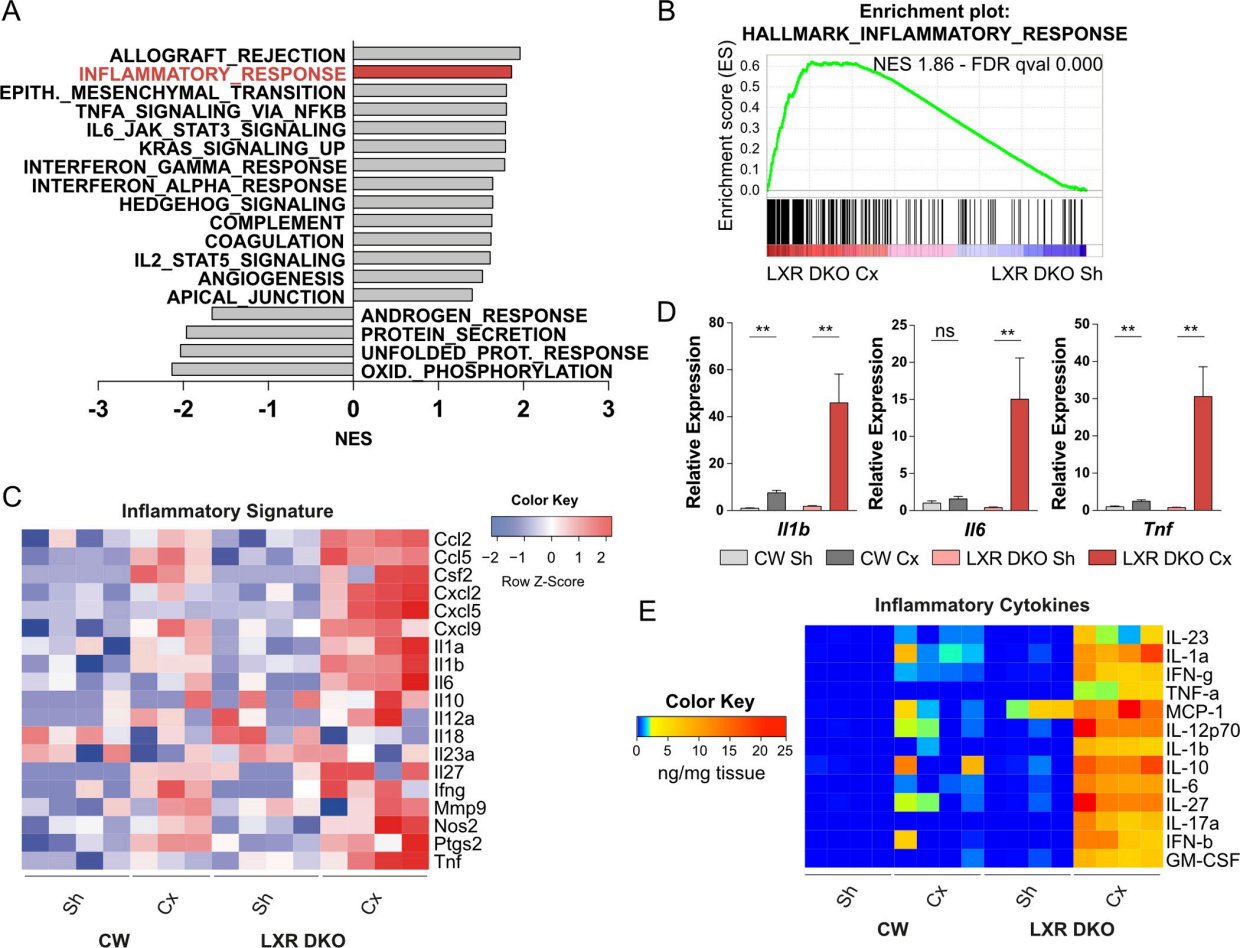
Androgen deprivation promotes chronic inflammation in LXR-null prostates. (A and B) Hallmark gene sets enrichment scores (A) and enrichment plot of inflammatory gene set (B) by GSEA analysis of RNA sequencing showing an inflammatory response following 1-month castration in LXR DKO. (C) RNA sequencing analysis of pro-inflammatory genes expression (log2FC) confirms a pro-inflammatory environment in 1-month Cx LXR DKO mice. (D) Inflammatory *Il1b*, *Il6*, and *Tnf* gene expression analysis by RT-qPCR. (E) Quantification of intraprostatic inflammatory cytokines by multiplex beads-based immunoassay. Data are expressed in nanograms per micrograms of tissue. Groups are composed of at least 4 animals. Bars represent mean ± SEM. Statistical analyses were performed via Mann–Whitney test. *p < 0.05, **p < 0.01, ***p < 0.001 and ns. For numerical raw data, please see S1 Data. For supporting dataset, please see S2–S6 Datas. CW, control wild-type; Cx, castrated; LXR, liver X receptor; GSEA, Gene Set Enrichment Analyses; LXR DKO, LXR alpha and beta double knock-out; NES, Normalized Enrichment Score; ns, nonsignificant; RT-qPCR, quantitative reverse transcription PCR; Sh, sham-operated.

To further evaluate the impact of phagocytosis-deficient macrophages accumulation, we performed further GSEA analyses using “hallmarks” gene sets. This revealed significant positive enrichment of immunity-related gene sets as well as an expected significant negative enrichment of androgen response and protein secretion gene sets in LXR DKO prostates in response to 1-month castration compared to sham operation (Fig 5A and S5 and S6 Datas). Interestingly, inflammatory response was one of the most positively enriched gene sets (Fig 5B). Consistent with this, analysis of a curated inflammatory gene signature in RNA sequencing data showed a marked up-regulation, which was restricted to LXR DKO castrated prostates (Fig 5C). Increased expression of major inflammatory cytokines *Il1b*, *Il6*, and *Tnf* was confirmed by quantitative reverse transcription PCR (RT-qPCR) (Fig 5D). This was further confirmed by multiplex assays, which showed increased production of inflammatory cytokines in response to 1-month castration in LXR DKO prostates (Fig 5E). The origin of inflammatory signature could result from LXR-deficient macrophages as well as myeloid and lymphoid infiltration (Fig 2E). Altogether, these results demonstrated that impaired phagocytosis of apoptotic cells induced by androgen deprivation was correlated with the development of chronic inflammation of the prostate.

### Osteopontin inflammatory cytokine produced by LXR-deficient macrophages stimulates proliferation of epithelial prostatic cells

**Fig 6:**
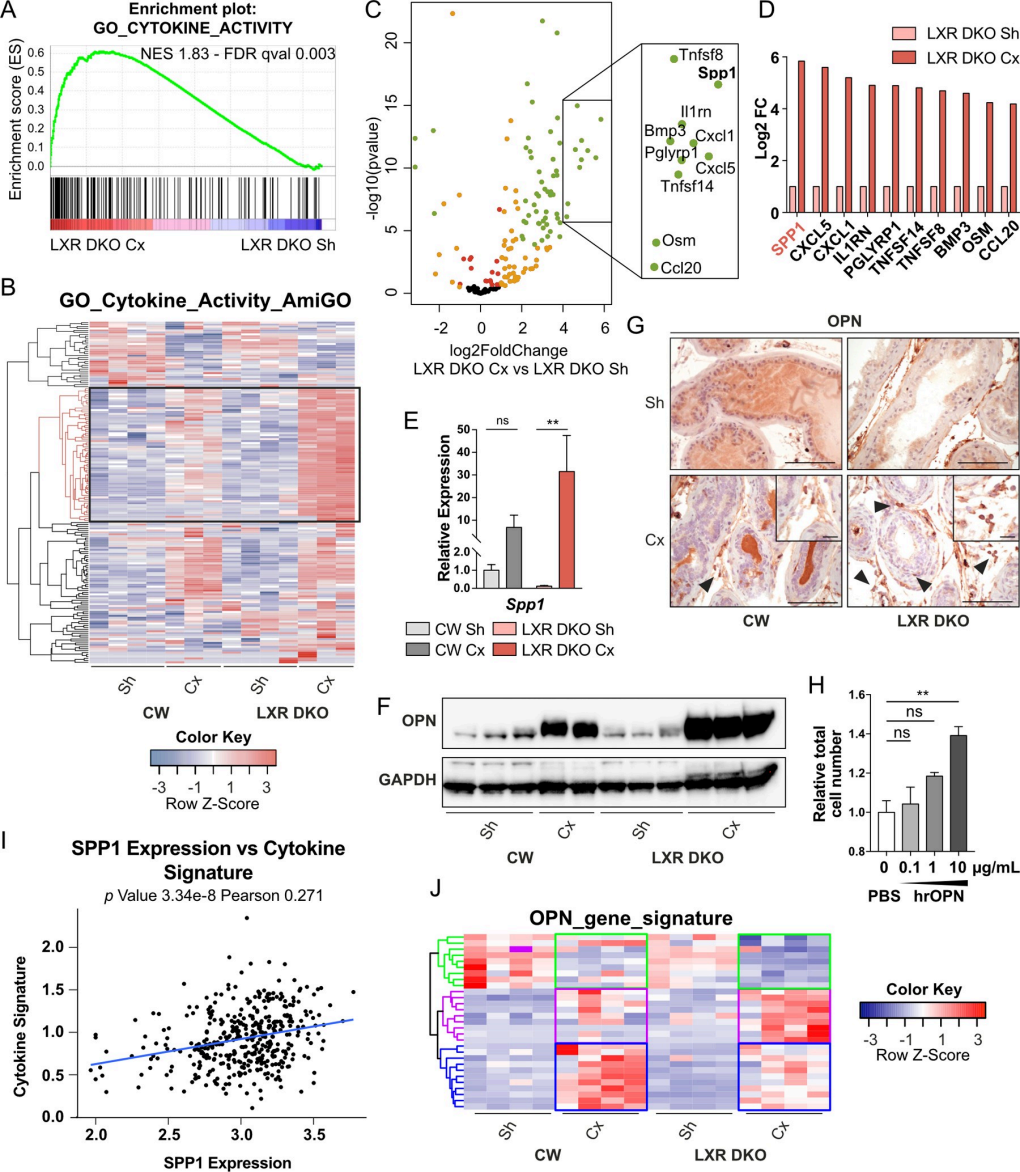
Inflammatory cytokine OPN is up-regulated in LXR-null prostate in response to androgen deprivation. (A) GO analysis of RNA sequencing reveals enrichment in genes annotated by the cytokine activity GO term GO:0005125 from MSigDB after 1 month of castration in LXR DKO mice in comparison with Sh mice. (B) RNA sequencing analysis of cytokine activity coding genes annotated by the GO term GO:0005125 according to AmiGO 2 annotation. (C) Volcano plot of cytokine activity coding genes shows differential gene expression in 1-month Cx LXR DKO compared with Sh LXR DKO mice. Most of the genes are statistically deregulated (padj < 0.05—red), with an abs(logFC) >1 (orange). Among them, a large number of genes are strongly up-regulated (abs(logFC) > 2, padj < 0.001—green). (D) Top 10 list of the most up-regulated genes in the 1-month Cx versus Sh LXR DKO mice. (E) RT-qPCR analysis of the most deregulated gene OPN coding gene *Spp1*, in response to 1-month castration in LXR DKO prostates. (F and G) Western blot analysis (F) and immunohistochemical detection (G) of OPN. OPN protein is expressed at high levels in prostate stroma and low levels in epithelial cells of LXR DKO mice, in response to 1-month castration. Groups are composed of at least 4 animals. Bars represent mean ± SEM. Statistical analyses were performed via Mann–Whitney test. Scale bars, 100 μm; insets, 20 μm. (H) P69 benign epithelial prostate cells were treated by increasing doses of hrOPN for 24 hours. A pro-proliferative effect was observed under stimulation by 10-μg/mL hrOPN. (I) Correlation plot between SPP1 expression and cytokine signature of LXR DKO mice using TCGA cohort [72] (J) Heatmap of OPN putative target genes [25], identification of 3 gene clusters differentially deregulated (green, purple, and blue). Bars represent mean of triplicates ± SEM. Statistical analyses were performed via Kruskal–Wallistest. *p < 0.05, **p < 0.01, ***p < 0.001 and ns. For numerical raw data, please see S1 Data. For raw immunoblots, please see S1 Blots. For supporting dataset, please see S2, S7 and S8 Datas. Cx, castrated; GO, gene ontology; hrOPN, human recombinant osteopontin; LXR, liver X receptor; LXR DKO, LXR alpha and beta double knock-out; ns, nonsignificant; OPN, osteopontin; Sh, sham-operated; RT-qPCR, quantitative reverse transcription PCR; SPP1, secreted phosphoprotein 1; TCGA, The Cancer Genome Atlas.

Given the central role of cytokine-mediated dialog between immune cells and adjacent epithelial cells to maintain tissue homeostasis, we characterized cytokine function in chronic inflammation observed in the context of androgen deprivation. GSEA analysis with gene ontology (GO) terms showed a significant positive enrichment of cytokine activity in LXR DKO castrated compared to sham-operated mice (Fig 6A). Extraction of gene expression from our RNA sequencing data demonstrated that a set of cytokines coding genes present in the AmiGO cytokine activity gene set (GO:0005125) were specifically increased in 1-month castrated LXR DKO prostates (Fig 6B and S7 Data). Interestingly, secreted phosphoprotein 1 (SPP1), which encodes the OPN cytokine, was the most deregulated (by Log Fold change) in LXR DKO compared to sham-operated mice (Fig 6C and 6D). This was consistent with data from the literature, showing that LXRs can indirectly down-regulate *SPP1* expression through activator protein 1 (AP-1) [22,23]. OPN deregulation was further confirmed by RT-qPCR analysis of *Spp1* gene expression (Fig 5E) and western blot analysis (Fig 6F). Immunohistochemical detection of OPN revealed that this cytokine was mostly produced by stromal cells and some cells localized within the myofibrillar layer surrounding acini (Fig 6G). To asses if stromal secretion of OPN could influence the epithelial compartment, we treated human benign epithelial prostatic P69 cells with increasing amounts of human recombinant OPN for 24 hours. Interestingly, OPN stimulated proliferation of non-tumor prostatic cells in a dose-dependent manner (Fig 6H). According to OPN status in prostate cancer as a bad prognosis marker [24], *SPP1* expression has been found overexpressed between non-tumoral, primary site and metastasis localization (S6A Fig). Moreover, SPP1 expression in human datasets correlated with inflammatory cytokine signature of LXR DKO mice identified in Fig 4C (Figs 6I and S4A). To further investigated OPN signaling pathway in LXR DKO mice, we analyzed expression of integrin genes encoded cognate OPN-receptors (S6B Fig) as well as OPN target genes identified in mammary carcinoma cells [25]. Thus, we identified 3 clusters of genes differentially deregulated between wild-type and DKO mice that could represent a potential significant OPN-signature driving epithelial cell proliferation (Fig 6J and S8 Data). Together, these observations suggested that the inflammatory cytokine osteopontin produced in the context of chronic inflammation induced hyperproliferation of adjacent epithelial cells.

### Androgen deprivation-induced chronic inflammation promotes hyperproliferation of epithelial cells and emergence of prostate neoplasia

**Fig 7:**
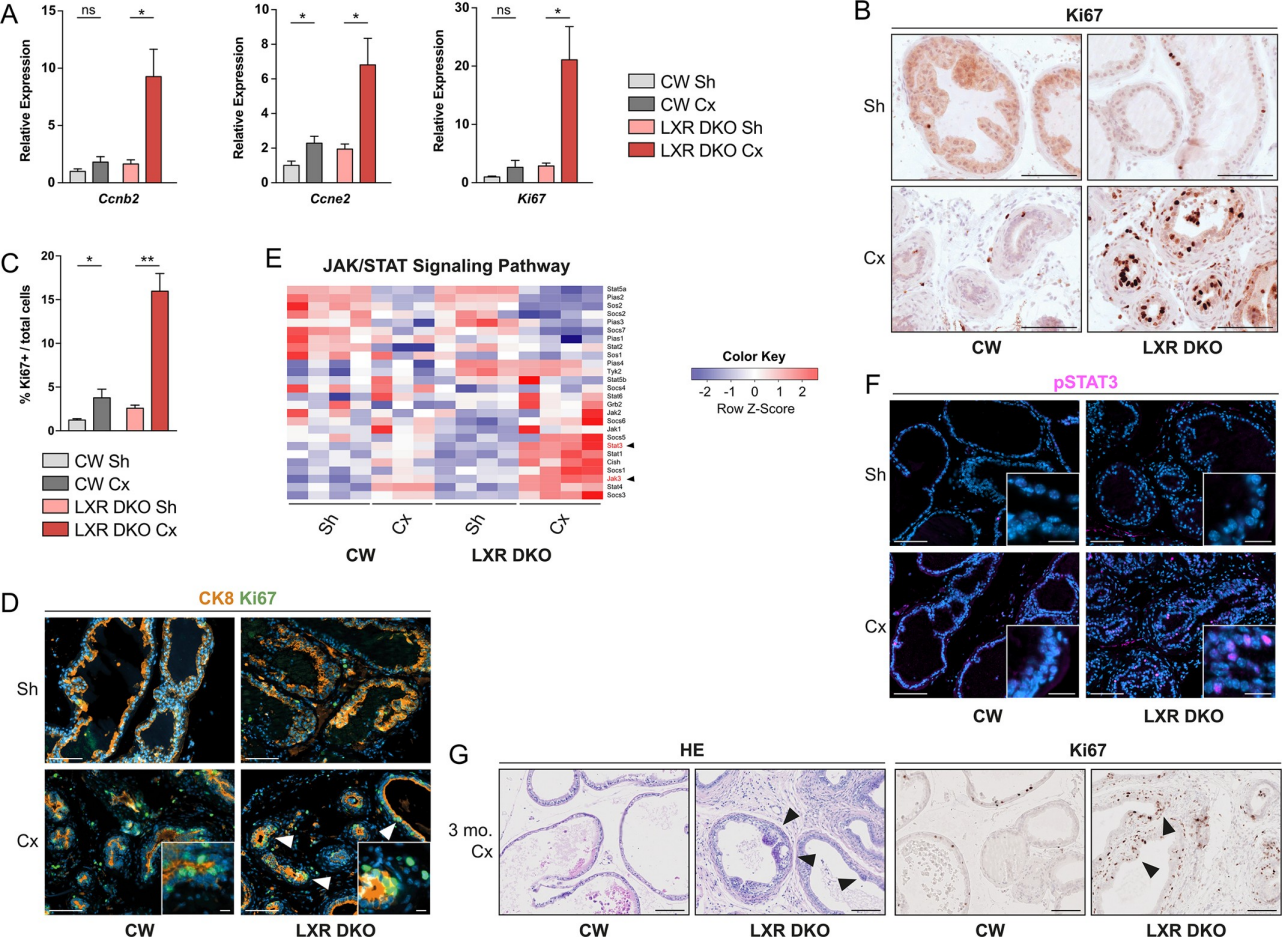
Castration-induced chronic inflammation promotes prostate neoplasia development in LXR-null mice. (A) RT-qPCR analysis of cyclins genes *Ccnb2* and *Ccne2* and *Ki67* proliferation marker gene. (B and C) Immunohistochemical detection of Ki67 proliferation marker (B) and proliferation index of prostate in Sh or 1-month Cx mice (C). (D) Proliferation marker Ki67 colocalization with epithelial cells marker CK8 demonstrates high proliferation capacities of 1-month Cx LXR DKO prostate. (E) RNA sequencing analysis of JAK/STAT signaling pathway genes expression. (F) Histological analysis of phospho-STAT3 in CW and LXR DKO prostates following 1-week castration. (G) After 3 months of castration, highly proliferative lesions seen in response of 1 month of castration in LXR-null prostates progress to neoplastic PIN lesions as demonstrated by hematoxylin and eosin staining and Ki67 immunohistochemical detection. Groups are composed of at least 4 animals. Bars represent mean ± SEM. Statistical analyses were performed via Mann–Whitney test. *p < 0.05, **p < 0.01, ***p < 0.001 and ns. Scale bars, 100 μm; insets, 10 μm. For numerical raw data, please see S1 Data. For supporting dataset, please see S2 Data. Cx, castrated; CW, control wild-type; JAK/STAT, Janus kinase/signal transducers and activators of transcription; LXR, liver X receptor; LXR, liver X receptor; LXR DKO, LXR alpha and beta double knock-out; ns, nonsignificant; PIN, prostatic intraepithelial neoplasia; RT-qPCR, quantitative reverse transcription PCR; Sh, sham-operated.

To further evaluate a potential role of inflammatory cytokines in modulating proliferation of epithelial cells in vivo, we analyzed the expression of proliferation markers by RT-qPCR (Fig 7A). This showed a dramatic increase in expression of *CyclinB2*, *CyclinE2*, and *Ki67* following 1 month of castration in LXR DKO mice, whereas castration had little to no impact in CW prostates (Fig 7A). Immunohistochemical detection of Ki67 further confirmed a specific increase in proliferation rate in response to 1 month of castration in LXR DKO but not CW mice (Fig 7B and 7C). Hyperproliferative cells were mostly epithelial cells as confirmed by colocalization of Ki67 proliferation with the epithelial cell marker CK8 (Fig 7D). Among pathways deregulated by castration in LXR DKO mice, GO analysis showed altered IL6/JAK/STAT3 signaling (Fig 5A). IL6/JAK/STAT3 axis is known to drive proliferation in prostate cancer cells [26]. Thus, we wondered if STAT3 signaling could be up-regulated. Detailed analysis of the JAK/STAT pathway showed that JAK3 and STAT3 were the most up-regulated in response to 1-month castration in LXR DKO prostates (Fig 7E). We further confirmed a large increase in phospho-STAT3 nuclear staining in epithelial cells of LXR DKO castrated prostates (Fig 7F). Subsequently, to determine if hyperproliferation of epithelial cells could initiate tumor development, we performed 3- and 6-month castrations in CW and LXR DKO mice. Although long-term castration had little to no impact in CW mice, it resulted in the development of Ki67^+^ hyperproliferative, neoplastic lesions in LXR DKO mice (Fig 7G). Altogether, these data showed that chronic inflammation resulting from aberrant accumulation of phagocytosis-deficient macrophages in LXR DKO prostates was associated with STAT3 signaling deregulation and could result in epithelial neoplasia.

## Discussion

Cancer-related inflammation can contribute to both cancer initiation and progression. While chronic inflammation is a well-known risk factor for some cancers like colon, liver, or lung, the causal relationship for prostate cancer is more debated. Here, we show that chronic inflammation can be the result of impaired apoptotic cells clearance by macrophages in response to androgen deprivation in mice deficient for LXRs, key sensors of cholesterol homeostasis. We further show that this state of chronic inflammation, through the cytokine OPN, can cause aberrant proliferation of prostate epithelial cells and, eventually, tumor initiation. This robust pro-inflammatory effect of castration, associated with blunted prostate regression, is mostly observed in mice deficient for LXRs. This suggests a strong interaction between deregulation of cholesterol metabolism and the effects of androgen deprivation, the mainstay of treatment, since the demonstration by Huggins and Hodges of the androgen dependence of the prostate and the efficacy of castration to induce tumor regression [27].

This interaction could be the result of a more or less direct effect of LXRs ablation on AR stability and/or activity. However, our data show no difference in expression or localization of AR within the prostate of LXR DKO mice compared with CW mice. Moreover, validated AR target genes as well as androgen-responsive genes (defined by our RNA sequencing analyses) respond in a similar manner to castration, independently of LXR status. In contrast, we demonstrate that castration in LXR DKO prostates is associated with robust recruitment of immune cells, in particular macrophages. Interestingly, some studies in patients highlighted that androgen deprivation not only induced regression of prostate gland but also caused profound alterations in immune microenvironment. Indeed, ADT induces prostate cancer infiltration by immune cells, predominantly characterized as CD3^+^, CD4^+^, and CD8^+^ T cells and CD68^+^ macrophages [16–18,28]. How these cells are recruited is unclear. One hypothesis is that androgens/AR signaling could directly modulate both innate and adaptive immune systems. Indeed, AR is expressed by macrophages, and multiple studies have demonstrated involvement of androgens/AR signaling in regulation of cytokine production and inflammation [29]. A second hypothesis for increased T cell infiltration is that epithelial cells that undergo apoptosis following ADT [30,31] could represent a source of antigens favoring APC recruitment and the subsequent activation of T cells, which would eventually result in clearance of apoptotic cells by macrophages [32–34]. The release of antigens in response to androgen deprivation could also be the result of some other forms of programmed cell death such as necroptosis, which is known to initiate an inflammatory response [35]. Here, we demonstrate that LXR DKO mice present an impaired phagocytosis activity toward prostate apoptotic cells. This is consistent with data showing that LXRs are critical for apoptotic cell clearance and maintenance of immune tolerance following phagocytosis in a model of autoimmune glomerulonephritis [36]. This effect is dependent on the LXR target gene *Mertk*, a tyrosine kinase receptor involved in the recognition of apoptotic cells through the expression of phosphatidyl serine-bound Gas6 opsonin, exposed at the outer cell membrane, i.e., a “eat-me” signal for phagocytes [37]. Interestingly, analysis of our RNA sequencing data shows decreased expression of *Mertk* in LXR DKO 1-month castrated mice compared to CW mice, suggesting that this may also participate in decreased phagocytic activity in the prostate. Another study demonstrated that LXR-dependent retinoic acid receptor alpha (RARα) transcriptional activity could enhance phagocytosis of apoptotic cells by macrophages via the expression of TGM2, a key factor for macrophage phagocytosis [38]. Whether this mechanism also plays a role in the prostate remains to be determined.

Our data relying on RNA sequencing and cytokine arrays clearly demonstrate that LXR DKO prostates develop a state of chronic inflammation following castration. In a normal situation, efficient phagocytosis of dead cells is accompanied by a switch from a pro-inflammatory to a immunosuppressive environment associated with production of tolerogenic cytokines transforming growth factor beta (TGFβ) and interleukin 10 (IL-10) (reviewed in [39]). This process of resolution has been shown to be impaired in LXR DKO macrophages [36]. In the case of LXR DKO prostates, deficient phagocytosis would result in maintenance of a pro-inflammatory status, associated with increased recruitment of immune cells (in particular, phagocytes) to try to overcome deficient clearance of apoptotic cells. The phenomenon would then self-amplify through secretion of further pro-inflammatory cytokines by newly recruited cells. Interestingly, data from the literature demonstrate that LXRs also exert a direct role as endogenous inhibitors of inflammatory response in macrophages. Indeed, LXR activation both in vitro and in vivo has been shown to down-regulate the production of pro-inflammatory cytokines such as IL-1β, Il-6, and tumor necrosis factor alpha (TNFα) in macrophages under lipopolysaccharide (LPS) or bacterial stimulation and in models of contact dermatitis, atherosclerosis, lung inflammation, and neuroinflammation [11,12,14,15,40,41]. LXRs have also been shown to inhibit OPN expression in macrophages using a crosstalk with AP-1 transcription factor leading to decrease promoter transcriptional activity [22,23]. Consistent with these observations in other tissues, our data show a massive increase in accumulation of IL-1β, Il-6, TNFα, and OPN in LXR DKO prostates. However, in this case, pro-inflammatory cytokines accumulation is triggered by castration rather than classical inflammatory stimuli. Chronic inflammation resulting from bacterial or viral infections [3] has been proposed to play a role in prostate cancer development, although a causal link has not been established [7]. Interestingly, our data show that sterile chronic inflammation in LXR DKO prostates following castration induces a massive accumulation of the cytokine OPN and development of prostate neoplasia. In line with the literature [42,43], we further show that OPN is able to stimulate proliferation of normal prostate cells in culture. Interestingly, OPN has been associated with proliferation and progression of prostate cancer in a mouse model in which its expression gradually increased from early neoplasia to aggressive cancer stages [44]. In patients, OPN is expressed by cancer cells as well as macrophages. Its expression increases together with Gleason score and could be associated with poor response to therapy [45–48]. Consistent with this, our analysis of OPN expression in publicly accessible prostate cancer cohorts confirmed its association with prostate cancer progression (SI Appendix S5B Fig). Moreover, survival was significantly reduced for patients who showed high levels of OPN [24]. Interestingly, *SPP1* expression was also correlated with inflammatory cytokine signature (Fig 6I). Taken together, these observations and our data strongly suggest that OPN could be one of the key mediators of the pro-neoplastic effect of castration in LXR DKO prostates and that it may also be involved in prostate cancer (PCa) progression in patients through deregulated inflammation.

Prostate cancer incidence is higher in western countries, and this has been suggested to result from higher incidence of metabolic syndrome and obesity. However, despite an abundant literature on meta-analyses, there is still no clear correlation between metabolic syndrome and prostate cancer in patients [7–10]. Data obtained from studies on mouse models demonstrate that a high-fat diet induces a systemic low burden inflammation concomitant with activation of NF-κB pathway and production of cyclooxygenase-2 (COX2) and inducible nitric oxide synthase (iNOS) inflammatory factors. This results in a STAT3/NF-κB-dependent inflammation of the prostate gland [5,49]. Interestingly, another study shows that inflammation of the prostate, induced in mice fed a high-fat diet, is associated with the development of neoplastic lesions [50]. Moreover, high-fat diet–induced inflammatory cytokines secretion by prostatic macrophages has been associated with prostate cancer progression [51,52]. Our data relying on gene ablation of key regulators of lipid metabolism have previously demonstrated that a high-cholesterol diet was sufficient to induce development of neoplasia in LXR DKO prostates, showing that alterations of cholesterol metabolism within the prostate can initiate prostate precancerous lesions [19]. Here, we show that castration, as a model of ADT, recapitulates both development of chronic inflammation of the prostate and the initiation of neoplasia in the absence of LXR. This shows that androgen deprivation exacerbates prostate inflammation induced by metabolic disorders, either as a consequence of a lipid-rich diet or cholesterol homeostasis deregulation (following LXR ablation) and suggests that this phenomenon could be relevant for prostate cancer patients with metabolic syndrome condition. Consistent with this, the response rate to ADT is affected in obese prostate cancer patients [53]. Furthermore, patients with metabolic syndrome present a shorter time to biochemical progression and thus develop lethal castration-resistant pathology earlier than patients without metabolic syndrome [54]. As a consequence, mCRPC patients with metabolic syndrome present a shorter progression-free survival than patients without metabolic syndrome [55]. However, whether this relies on chronic prostate inflammation following ADT in this disturbed metabolic context remains to be determined. Interestingly, there is abundant literature showing that ADT itself increases the risk to develop a metabolic syndrome [56–58]. Together with our data, this suggests that a self-sustaining loop between metabolic syndrome and ADT may be responsive for inflammation-mediated tumor recurrence.

Altogether, our results demonstrated that impaired macrophages response to androgen deprivation promotes chronic inflammation and further development of neoplastic lesions in the prostate of mice deficient for LXR, key transcriptional regulators of cholesterol homeostasis. This raises the question of ADT-triggered inflammation in patients, in particular in a context of cholesterol homeostasis deregulation.

## Materials and methods

### Ethics statement

All experiments were approved by Auvergne Ethics committee (CEMEAA) and registered according the approval number 7579–2016111416497784 V2.

### Animals

*Nr1h3* ^*−/−*^: *Nr1h2*^*−/−*^ referred to as LXR DKO mice as well as *Nr1h3* ^loxp/*loxp*^: *Nr1h2*^*loxp/loxp*^ were obtained from Dr. David Mangeldorf’s Lab (Department of Pharmacology and Biochemistry, University of Texas Southwestern Medical Center, Dallas, Texas). *Nr1h3* ^loxp/*loxp*^: *Nr1h2*^*loxp/loxp*^ were mate with Pb-Cre4 mice [59] in order to allow specific prostatic epithelial cells recombination of LXRs; Pb-Cre4; *Nr1h3* ^loxp/*loxp*^: *Nr1h2*^*loxp/loxp*^ referred to as *Lxrαβ* ^*pe-/-*^. Control wild-type (CW) are LXR sufficient (*Nr1h3*^*+/+*^:*Nr1h2*^*+/+*^*)* mice. All mice were maintained on a mixed background mostly composed of C57BL/6J and 129. All mice were castrated or sham operated at 4 months of age for 1 week, 1 month, 3 months, or 6 months and culled by cervical dislocation. Prostates were either frozen in liquid nitrogen or fixed in 4% paraformaldehyde. Total mRNAs were extracted using RNAII nucleotide extraction kit (Macherey Nagel, Düren, Germany) according to manufacturer’s instructions. Mice used for phagocytosis assays were euthanized at 2 months of age by CO_2_ inhalation.

### RNA sequencing analysis

RNA sequencing libraries were prepared using the Directional mRNA-Seq Sample Prep with polyA selection (Illumina, San Diego, California) following the manufacturer’s protocol. Single-end sequencing Illumina HiSeq 4000 was performed by the GenomEast platform (ANR-10-INBS-0009). The quality of reads was checked using FASTQC (v0.11.8). Filtered out reads correspond to reads with length lower than 40 bases after adapter and low bases quality removal. Reads were mapped onto the mm10 version of the *Mus musculus* genome using STAR version 2.5.3a. Normalized coverage was obtained by geneBodyCoverage tool from RSeQC version 2.6.4. Quantification of gene expression was performed using HTSeq version 0.6.1p1 with annotations coming from Ensembl version 90. Differential gene expression analysis was performed with DESeq2 package (v1.24.0). Comparisons of interest were performed using the statistical method proposed by Anders and Huber [60]. *p*-Values were computed using the Wald test and adjusted for multiple testing using the Benjamini and Hochberg method [61]. A gene identified as differentially expressed has an adjusted *p*-value lower than 0.05 and an absolute log2 Fold Change (logFC) value greater than 1.00. All RNA sequencing analyses were performed by R studio (v1.0.136). Heatmaps were generated using R « gplots » v3.0.1. and “pheatmap 1.0.12” libraries. Dataset is available using GEO accession number GSE134137.

### Gene set enrichment analyses

Gene set enrichment analysis were conducted using GSEA 3.0 [62] with MSigDB gene sets. GSEA was run in preranked mode using 1,000 permutations. Genes lists were ranked on the basis of log2 FC between the different experimental conditions.

### RT-qPCR

A total of 300 nanograms of total mRNAs from prostate tissues were reverse transcribed for 1 hour at 37°C with 5 pmoles of random hexamer primers, 200 units reverse transcriptase (MMLV RT, M1701, Promega, Madison, Wisconsin), 2 mM dNTPs, and 20 units RNAsin (N2615, Promega). Two microliters of a one-tenth dilution of cDNA was used in each quantitative PCR. PCR reactions were conducted with SYBR qPCR Premix Ex Taq II Tli RNase H+ (TAKRR820W, Takara, Saint-Germain-en-Laye, France). Primer pairs are listed in SI Appendix S1 Table. For each experiment and primer pairs, efficiency of PCR reactions was evaluated by amplification of serial dilutions of a mix of cDNAs. Relative gene expression was obtained by the ΔΔ*Ct* method with normalization to expression of *36b4* housekeeping gene.

### Histological analysis

Paraffin-embedded tissue sections were sectioned for hematoxylin and eosin staining. Alternatively, immunohistochemistry was performed on paraffin-embedded tissues after antigen retrieval if necessary, by boiling for 20 minutes in sodium citrate 10 mM, Tween 0.05% (pH 6); Tris 10 mM, EDTA 1 mM (pH 9.0); or Vector Unmasking Solution (H3300, Vector Laboratories, Burlingame, California), depending on the primary antibody. After 2.5% normal horse serum blocking for 1 hour, slides were incubated overnight at room temperature, with primary antibodies at the indicated concentrations (SI Appendix S2 Table). Primary antibodies were detected with appropriate polymers (ImmPress Polymer Detection Kit, Vector Laboratories). Polymer-coupled HRP activity was then detected with either Vectastain ABC (PKD4000, Vector Laboratories) for brightfield images or Tyramide SuperBoost Kits with Alexa Fluor Tyramide for fluorescence (Invitrogen, Waltham, Massachusetts). Nuclei were counterstained with hematoxylin or Hoechst (Invitrogen). Images were acquired with a Zeiss Axioplan 2, Zeiss AxioImager with Apotome2, or Zeiss Axioscan Z1 slide scanner (Zeiss, Oberkochen, Germany). They were minimally processed for global levels and white balance using Zeiss Zen (Zeiss). Image settings and processing were identical across genotypes.

### Western blot analysis

Proteins were extracted from tissues and cells using a high-salt buffer solution with Hepes 25 mM, EDTA 0.2 M (pH 8), MgCl_2_ 1.5 mM, NaCl 0.4 M, Nonidet-P40 1% supplemented with NaF 1 mM, Na_3_VO_4_ 1 mM, phenylmethylsulfonyl fluoride 1 mM, and complete protease inhibitor cocktail (Roche, Bâle, Switzerland). A total of 40 μg of total proteins were loaded on 4% to 12% Invitrogen NuPAGE Bis-Tris protein precast polyacrylamide gels and transferred onto Trans-Blot Turb Mini PVDF membranes. Membranes were incubated overnight at 4°C with primary antibodies either with 5% non-fat dry milk or BSA. Primary antibody detection was performed using peroxidase-conjugated anti-rabbit or anti-mouse antibodies (Abliance, Compiègne, France) and Clarity or Clarity Max Western ECL Blotting Substrates (Bio-Rad). Antibodies used for western blots are listed in SI Appendix S3 Table.

### Cytokines quantification by multiplex assay

Prostates were lysed in TER buffer (pH 7.4) (Tris 50 mM, EDTA 5 mM, and NaCl 250 mM) supplemented with NaF 1 mM, Na_3_VO_4_ 2 mM, phenylmethylsulfonyl fluoride 1 mM, and complete protease inhibitor cocktail (Roche). Cytokines quantification from prostate lysates was then performed using LEGENDplex Mouse Inflammation Panel (13-plex) bead-based immunoassay (BioLegend Ref. 140150) according to manufacturer’s instructions. Data were acquired on an LSRII flow cytometer (BD Biosciences, San Jose, California) and analyzed using LEGENDplex data analysis software v8.0.

### Flow cytometry

Single-cell suspensions of whole prostates were obtained from mice after digestion in HBSS medium (Invitrogen) containing 1.25 mM CaCl2, 0.4 mM MgSO4, 1 mg/mL DNase I (Roche), and 1 mg/mL type II collagenase (Sigma-Aldrich, Saint-Louis, Missouri) for 15 minutes at 37°C under gentle shaking. Digested tissues were washed with PBS supplemented with 2.5 mM EDTA, 0.1 mg/mL DNase I, and 0.5% BSA, and filtered through 50 μm filters (BD Medimachine from BD Biosciences).

Viability was assessed using the LIVE/DEAD Fixable Near-IR Dead Cell Stain Kit (Invitrogen) according to the manufacturer’s instructions. Cells were then washed and incubated in a blocking buffer (PBS, 2.5 mM EDTA, 0.5% BSA, 5 μg/mL anti-CD16/32 (Biolegend, San Diego, California)) for 10 minutes at 4°C. Cells were incubated for 20 minutes at 4°C with combinations of conjugated anti-mouse antibodies or corresponding isotypes (SI Appendix S4 Table). Finally, cells were washed and kept at 4°C until processing. Data were acquired on an LSRII flow cytometer (BD Biosciences) and analyzed using the FACSDiva Software (v6.1.3, BD Biosciences). Cells were first selected based on their size (FSC) and granularity (SSC) characteristics. Doublets were excluded from the analysis, thanks to the FSC/SSC criteria. Finally, live cells were gated based on their negative staining for the viability marker before they were selected for the various markers tested. The percentages presented for each cell population are reported as positivity minus the corresponding isotype control. All FCS files are available on flowrepository platform (https://flowrepository.org/) using ID: FR-FCM-Z33J.

### In vivo phagocytosis assay

In vivo phagocytosis assays were carried out as described [36]. Briefly, cell suspensions from whole prostates of 4 days castrated CW or LXR DKO donor mice were labeled with CFSE Cell Tracker (Biolegend) according to the manufacturer’s recommendations. Apoptotic cells rate was determined by flow cytometry analysis of Annexin V positive cells (PerCP/Cy5.5 Annexin V, Biolegend), according to the manufacturer’s instructions. Injected cells presented an apoptotic rate of 45.8% for the CW pool and 52.6% for the LXR DKO pool. Of note, 1e10^6^-labeled prostate cells from CW donor mice were injected IP into 2 months CW receiver mice; cells from LXR DKO donor mice were injected into LXR DKO receiver mice. Mice were killed after 1 hour, and the peritoneal cavity was flushed with 5 mL cold HBSS (Invitrogen). A single-cell suspension of peritoneal flush was stained with APC-coupled anti-F4/80 (Biolegend Ref. 123116) to identify macrophages. Cells were analyzed on an Attune NxT Flow Cytometer with Attune NxT software v2.6 (Invitrogen). Phagocytic index was determined as double positive CFSE F4/80^+^ expressed in % CFSE-labeled cells.

### Cell culture

The P69 human prostate cell line was obtained through Dr. Frédéric Bost’s lab (C3M, Inserm U1065, France). P69 (passage 16 to 18) cells were cultured in 10% FBS RPMI medium (Invitrogen). Cells were treated with recombinant human OPN protein (R&D systems, 1433-OP-050/CF, Minneapolis, Minnesota) at concentrations of 0.1, 1, or 10 mg/mL or PBS as negative control for 24 hours. After trypsinization, cell suspension count was performed on an automated LUNA cell counter (Logos Biosystems, Villeneuve d’Ascq, France).

### Lipid extraction

Lipid extraction was performed according to the method of Bligh and Dyer [63] in the presence of not naturally occurring lipid species as internal standards. The following lipid species were added as internal standards: D7-FC, CE 17:0, and CE 22:0. Tissue homogenates representing a wet weight of 2 mg were extracted. Chloroform phase was recovered by a pipetting robot (Tecan Genesis RSP 150, Tecan, Männedorf, Switzerland) and vacuum dried. The residues were dissolved in either 7.5 mM ammonium acetate in methanol/chloroform (3:1, v/v) (for low mass resolution tandem mass spectrometry) or chloroform/methanol/2-propanol (1:2:4 v/v/v) with 7.5 mM ammonium formate (for high resolution mass spectrometry).

### Mass spectrometric analysis

The analysis of lipids was performed by direct flow injection analysis (FIA) using a triple quadrupole mass spectrometer (FIA-MS/MS; QQQ triple quadrupole) and a hybrid quadrupole-Orbitrap mass spectrometer (FIA-FTMS; high mass resolution).

The Fourier Transform Mass Spectrometry (FIA-FTMS) setup is described in detail in Höring and colleagues [64]. CEs were recorded in positive ion mode FTMS in range m/z 500 to 1,000 for 1 minute with a maximum injection time (IT) of 200 ms, an automated gain control (AGC) of 1*106, 3 microscans, and a target resolution of 140,000 (at m/z 200). Multiplexed acquisition (MSX) was used for the [M+NH4]+ of free cholesterol (FC) (m/z 404.39) and D7-cholesterol (m/z 411.43) for 0.5 minute acquisition time, with a normalized collision energy of 10%, an IT of 100 ms, AGC of 1*105, isolation window of 1 Da, and a target resolution of 140,000. Data processing details were described in Höring and colleagues using the ALEX software [65], which includes peak assignment and intensity picking. The extracted data were exported to Microsoft Excel 2010 and further processed by self-programmed Macros. Lipid species were annotated according to the proposal for shorthand notation of lipid structures that are derived from mass spectrometry [66].

### Oil Red O staining

Lipid staining was performed on cryosection with Oil Red O (Sigma-Aldrich, O0625) according to the Biological Stain Commission Procedure [67].

### Prostate cancer patients’ cohort’s analysis

Publicly available gene expression data [68–72] were analyzed using CancerTool platform [73] http://genomics.cicbiogune.es/CANCERTOOL/index.html. “Violin” plots depicting the expression of SPP1 were generated to compare expression of SPP1 in non-tumoral (N), primary tumor (PT), and metastatic (M) PCa specimens. Statistical analyses were performed by ANOVA (multiple groups). Correlation plot for cytokine signature was generated using R library “ggplot2_3.1.1.” Pearson Correlation rho scores and *p*-values were obtained using R package « Hmisc_4.2–0 ».

### Statistical analysis

Data are expressed as mean ± SEM. Statistical analyses were performed with Mann–Whitney test except for proliferation assay of P69 cell line, which was performed with Kruskal–Wallis test. Values of *p* < 0.05 were considered significant. Statistical analyses were performed with GraphPad Prism Software v7.0a.

## Supporting information

S1 Fig Representative macroscopic observation of Sh and 1-month Cx mice shows a drastic regression of seminal vesicles (SV). AP, anterior prostate; BL, bladder; Cx, castrated; Sh, sham-operated; SV, seminal vesicle; VP, ventral prostate.
(TIFF)

S2 Fig Flow cytometry gating strategy identification of immune cells. CD45^+^ immune cells were defined as follows: CD4+ T4 lymphocytes, CD19+ B cells, CD11b^+^ Ly6C^−^ Ly6G^−^ F4/80^+^ SCC^low^ MOs, CD11b^+^ Ly6C^−^ Ly6G^−^ F4/80^−^ and CD11b^-^ CD11c^-^ F4/80^+^ other MPs, and CD11b^+^ Ly6C^−^ Ly6G^+^ neutro. DCs, dentritic cells; MOs, macrophages; MPs, mononuclear phagocytes; neutro, neutrophils.
(TIFF)

S3 Fig (A) Immunohistological staining of pan-leukocytes marker CD45 shows no increased immune cells infiltration in response to 1-month castration of Lxrαβ pe-/- in comparison with CW mice. Epithelial ablation of LXR did not induce obvious histological alterations after staining (B) or differential regression of dorsal lobes in response to castration (C). Groups are composed of at least 6 animals. Bars represent mean ± SEM. Statistical analyses were performed via Mann–Whitney test. *p < 0.05, **p < 0.01, ***p < 0.001 and ns. Scale bars, 100 μm. CW, control wild-type; Cx, castrated; DP, dorsal prostate; HE, hematoxylin eosin; LXR, liver X receptor; ns, nonsignificant.
(TIFF)

S4 Fig Gene ontology from the biological process subcollection analysis of RNA sequencing reveals that the 4 first enriched gene sets are related to leukocytes migration and chemotaxis after 1 month of castration in LXR DKO compared to Sh LXR DKO mice. For supporting dataset, please see S2 Data. Cx, castrated; ES, enrichment score; FDR, false discovery rate; LXR DKO, LXR alpha and beta double knock-out; NES, normalized enrichment score; Sh sham-operated.
(TIFF)

S5 Fig Flow cytometry gating strategy identification of CFSE-positive cells among F4/80 positive cells relative to the in vivo phagocytosis experiment. The determination of the percentage of CFSE+ / F4/80+ cells reveals that LXR DKO F4/80+ macrophages have reduced capacity to phagocytose CFSE-tracked cells (83.9% versus 95%). Bars represent mean ± SEM. Statistical analyses were performed via Mann–Whitney test. *p < 0.05, **p < 0.01, ***p < 0.001 and ns. For numerical raw data, please see S1 Data. APC, antigen-presenting cells; CFSE, carboxyfluorescein succinimidyl ester; CW, control wild-type; FSC, forward scatter; LXR DKO, LXR alpha and beta double knock-out; ns, nonsignificant; SSC, side scatter.
(TIFF)

S6 Fig (A) OPN expression is associated with prostate cancer progression in publicly available prostate cancer patients’ cohorts [68–72]. (B) Heatmap of genes encoded various OPN receptors. CW, control wild-type; Cx, castrated; OPN, osteopontin; Sh, sham-operated.
(TIFF)

S1 Blots Raw images. The file “S1_Blots” aggregates all uncropped and original western blots images.
(TIFF)

S1 Data Numerical raw data. All numerical raw data are combined in a single Excel file, “S1_Data.” This file consists of several spreadsheets. Each spreadsheet contains the raw data of 1 subfigure.
(XLSX)

S2 Data Prostates RNA sequencing ouput. RNA sequencing data from CW Cx versus CW Sh mice and LXR DKO Cx versus LXR DKO Sh mice. CW, control wild-type; Cx, castrated; LXR DKO, LXR alpha and beta double knock-out; Sh, sham-operated.
(XLSM)

S3 Data Identification of androgen-regulated gene set. ARG signature was established as genes differentially expressed in CW Cx versus CW Sh mice, with padj < 0.0001, log2FC <−2; >2. For numerical raw data, please see S1 Data. ARG, androgen-regulated genes; CW, control wild-type; Cx, castrated; log2FC, log2 fold change.
(XLSX)

S4 Data Definition of prostatic immune profile by flow cytometry analysis. Determination of absolute numbers of each immune cell population of WT mice Sham versus Castrated and LXR DKO mice Sham versus Castrated. LXR DKO, LXR alpha and beta double knock-out; WT, wild-type.
(XLSX)

S5 Data Lipid composition analysis of mouse prostates. Mass spectrometry analysis output from WT mice Sham versus Castrated and LXR DKO mice Sham versus Castrated. LXR DKO, LXR alpha and beta double knock-out; WT, wild-type.
(XLSX)

S6 Data Enrichment analysis of prostatic samples. Hallmark gene sets positive/negative enrichment results obtained by GSEA analysis of RNA sequencing in LXR DKO Cx compared to LXR DKO Sh mice. Cx, castrated; GSEA, Gene Set Enrichment Analyses; LXR DKO, LXR alpha and beta double knock-out; Sh, sham-operated.
(XLSX)

S7 Data Identification of inflammation signature. RNA sequencing data for framed genes related to Fig 6B, i.e., cytokine coding genes specifically upregulated in DKO 1-month castrated mice. DKO, double knock-out.
(XLSX)

S8 Data Identification of OPN-related signature. RNA sequencing data for OPN-target genes analysis related to Fig 6J specifically deregulated in DKO 1-month castrated mice. DKO, double knock-out; OPN, osteopontin.
(XLSX)

S1 Table Primers used for RT-qPCR. (DOCX)

S2 Table Antibodies and conditions used for immunohistochemical analyses. (DOCX)

S3 Table Antibodies used for western blot. (DOCX)

S4 Table Antibodies used for flow cytometry analysis. (DOCX)

## Abbreviations

ADT: androgen deprivation therapy
AGC: automated gain control
AP-1: activator protein 1
APC: antigen-presenting cells
APOE: apolipoprotein
AR: androgen receptor
ARG: androgen-responsive genes
CE: cholesteryl esters
CFSE: carboxyfluorescein succinimidyl ester
COX2: cyclooxygenase-2
FASN: fatty acid synthase
FC: free cholesterol
FIA: flow injection analysis
FTMS: Fourier Transform Mass Spectrometry
GSEA: Gene Set Enrichment Analyzes
iNOS: inducible nitric oxide synthase
IT: injection time
logFC: log2 Fold Change
LXR: liver X receptor
mCRPC: metastatic Castration-Resistant Prostate Cancer
MS: mass spectrometer
NF-kB: nuclear factor kappa B
OPN: osteopontin
PCa: prostate cancer
RARa: retinoic acid receptor alpha
RT-qPCR: quantitative reverse transcription PCR
SPP1: secreted phosphoprotein 1
