# Supplementary figures and images for: Absence of nuclear receptors LXRs impairs immune response to androgen deprivation and leads to prostate neoplasia
Source: PLoS Biol. 2020 Dec 7;18(12):e3000948. doi: 10.1371/journal.pbio.3000948 (PMC7752095; doi:10.1371/journal.pbio.3000948)

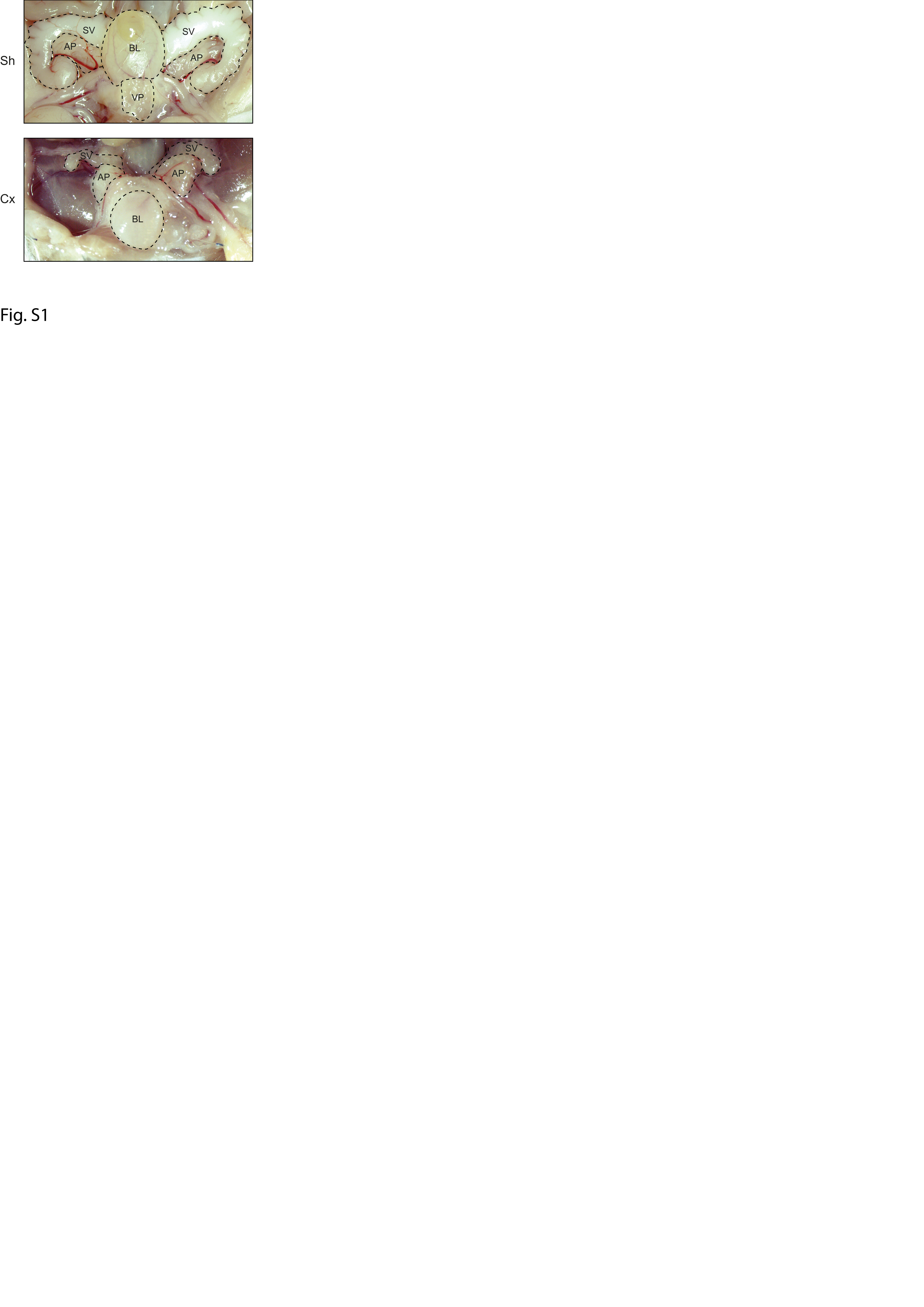

Supplement: S1 Fig — AP, anterior prostate; BL, bladder; Cx, castrated; Sh, sham-operated; SV, seminal vesicle; VP, ventral prostate. (TIFF) [file pbio.3000948.s001.tiff]

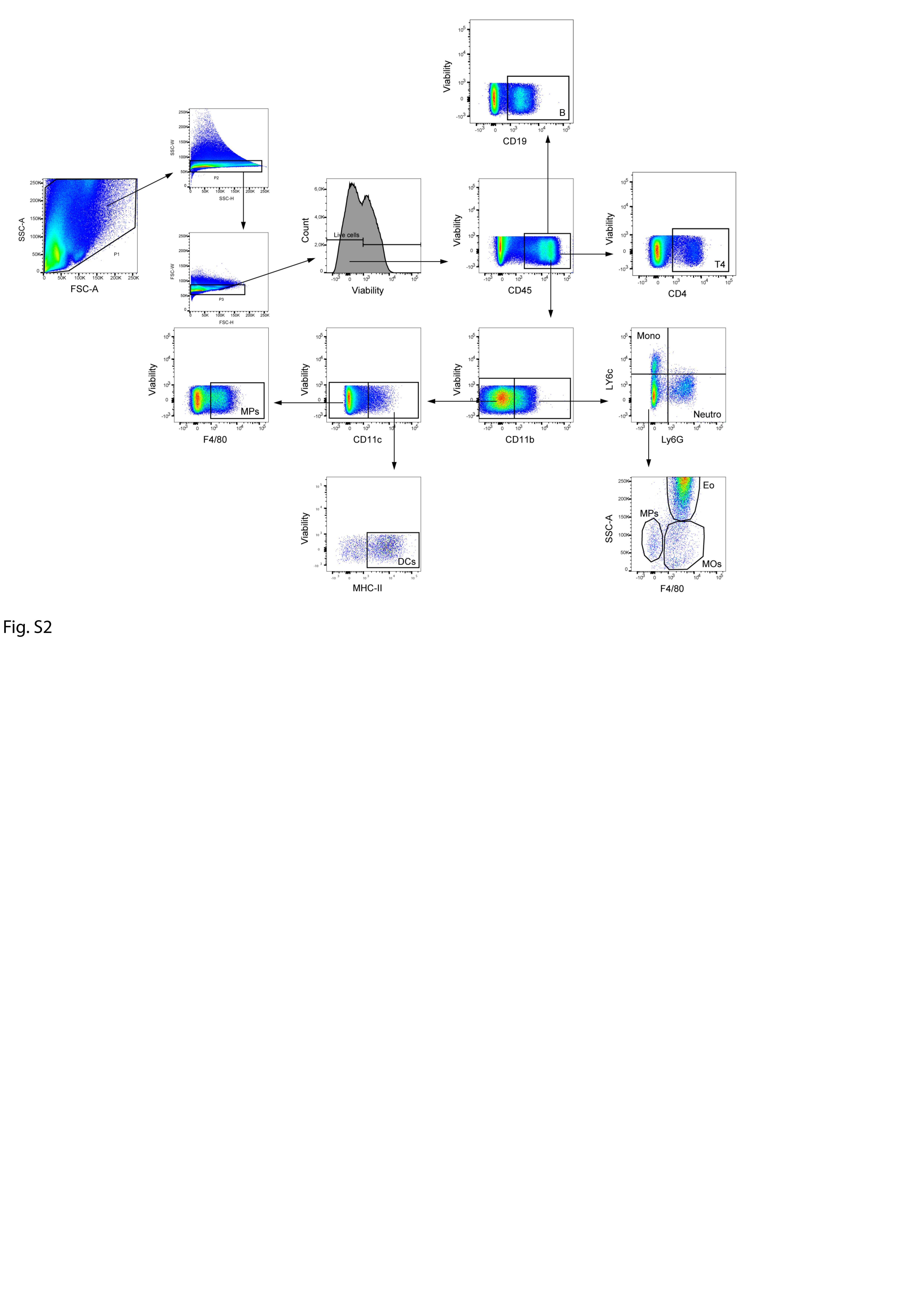

Supplement: S2 Fig — CD45+ immune cells were defined as follows: CD4+ T4 lymphocytes, CD19+ B cells, CD11b+ Ly6C− Ly6G− F4/80+ SCClow MOs, CD11b+ Ly6C− Ly6G− F4/80− and CD11b- CD11c- F4/80+ other MPs, and CD11b+ Ly6C− Ly6G+ neutro. DCs, dentritic cells; MOs, macrophages; MPs, mononuclear phagocytes; neutro, neutrophils. (TIFF) [file pbio.3000948.s002.tiff]

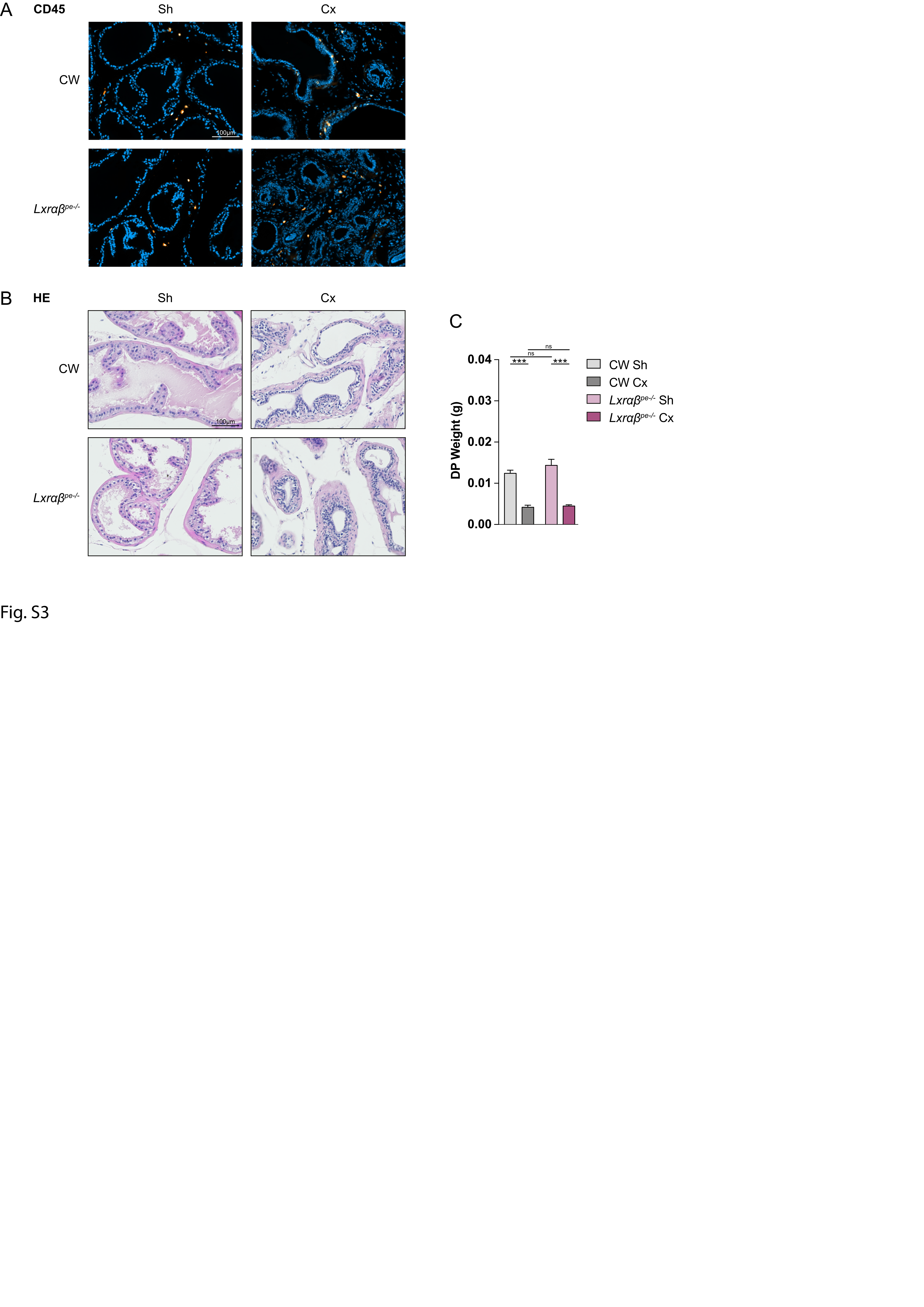

Supplement: S3 Fig — (A) Immunohistological staining of pan-leukocytes marker CD45 shows no increased immune cells infiltration in response to 1-month castration of Lxrαβ pe-/- in comparison with CW mice. Epithelial ablation of LXR did not induce obvious histological alterations after staining (B) or differential regression of dorsal lobes in response to castration (C). Groups are composed of at least 6 animals. Bars represent mean ± SEM. Statistical analyses were performed via Mann–Whitney test. *p < 0.05, **p < 0.01, ***p < 0.001 and ns. Scale bars, 100 μm. CW, control wild-type; Cx, castrated; DP, dorsal prostate; HE, hematoxylin eosin; LXR, liver X receptor; ns, nonsignificant. (TIFF) [file pbio.3000948.s003.tiff]

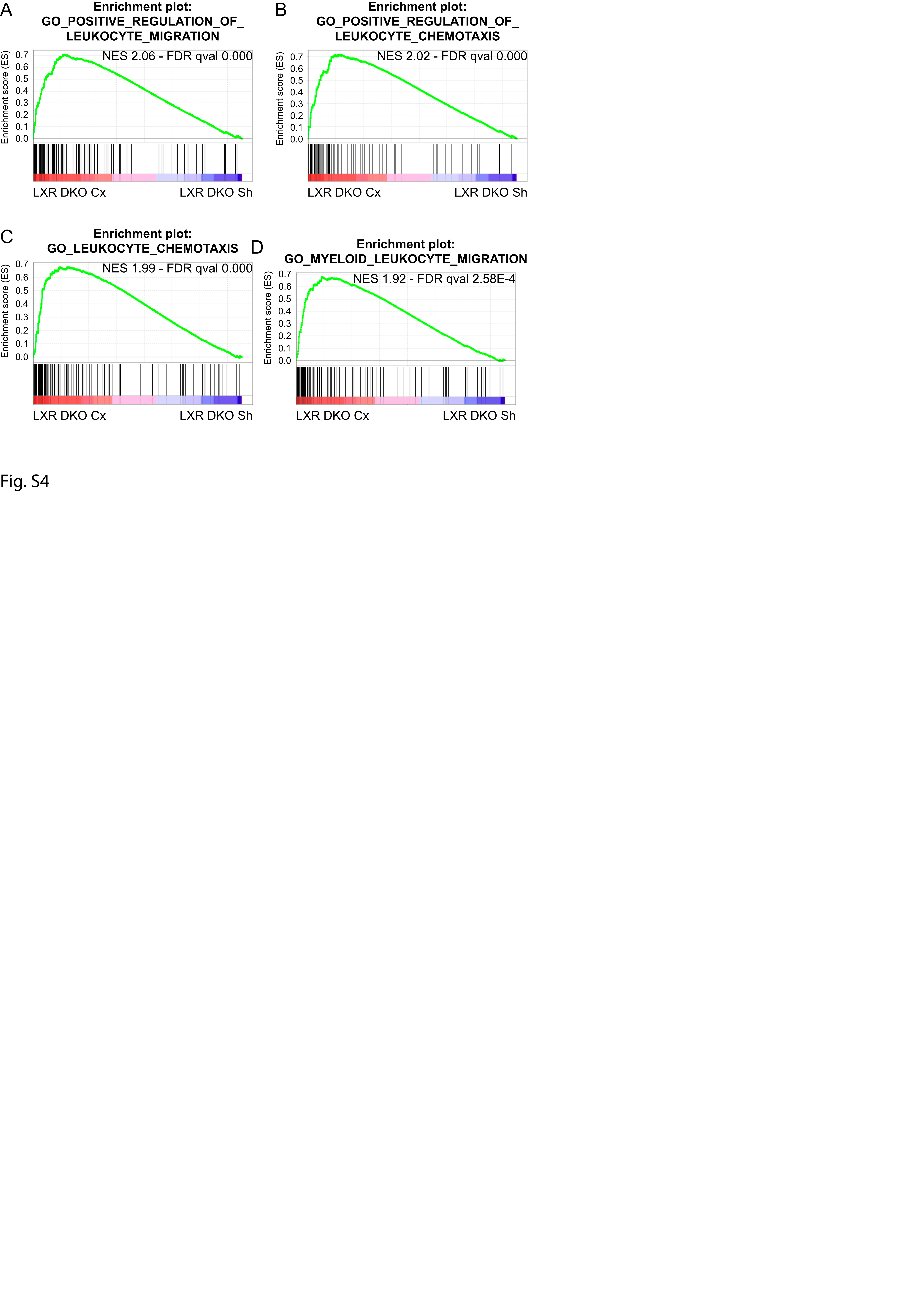

Supplement: S4 Fig — For supporting dataset, please see S2 Data. Cx, castrated; ES, enrichment score; FDR, false discovery rate; LXR DKO, LXR alpha and beta double knock-out; NES, normalized enrichment score; Sh sham-operated. (TIFF) [file pbio.3000948.s004.tiff]

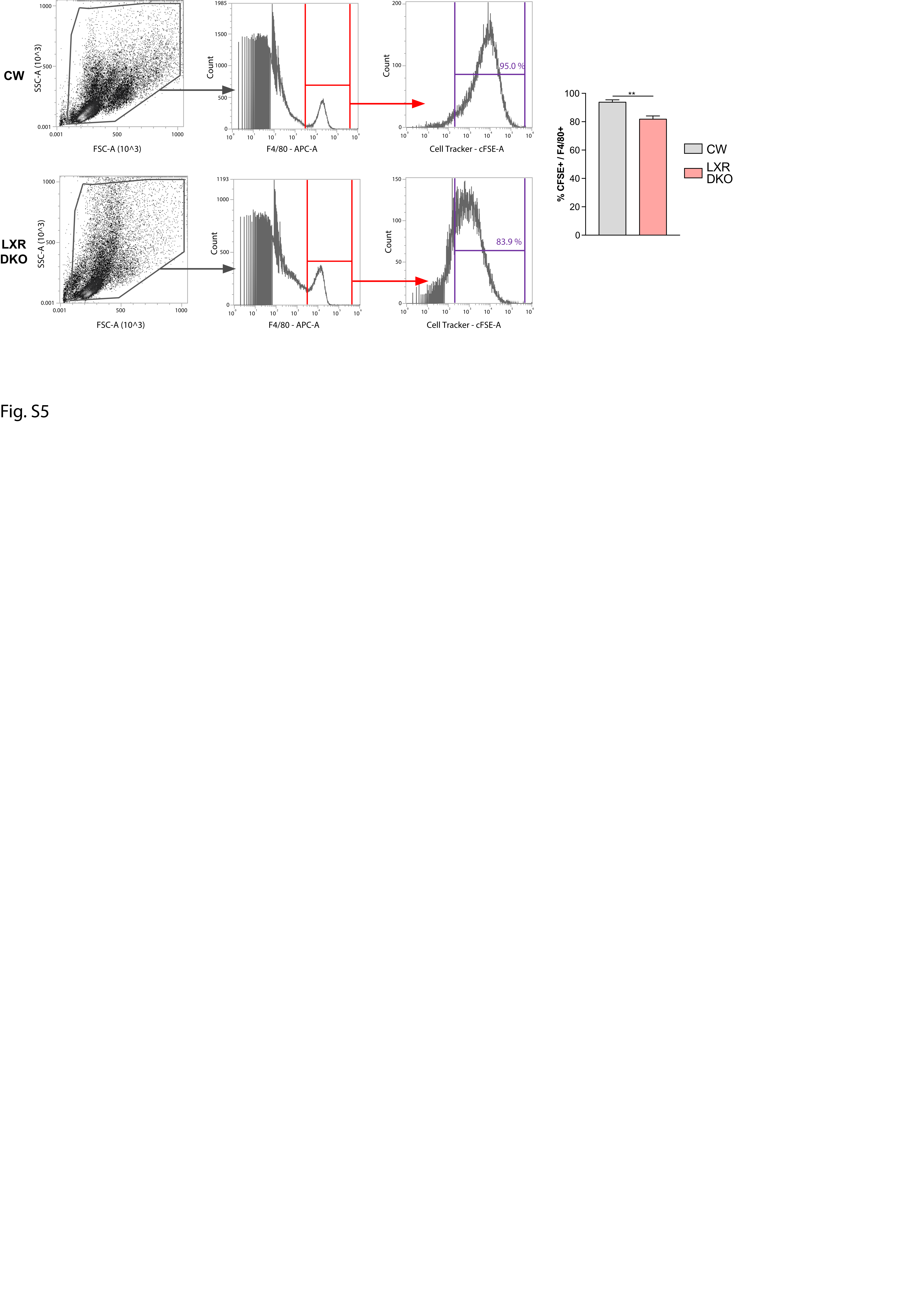

Supplement: S5 Fig — The determination of the percentage of CFSE+ / F4/80+ cells reveals that LXR DKO F4/80+ macrophages have reduced capacity to phagocytose CFSE-tracked cells (83.9% versus 95%). Bars represent mean ± SEM. Statistical analyses were performed via Mann–Whitney test. *p < 0.05, **p < 0.01, ***p < 0.001 and ns. For numerical raw data, please see S1 Data. APC, antigen-presenting cells; CFSE, carboxyfluorescein succinimidyl ester; CW, control wild-type; FSC, forward scatter; LXR DKO, LXR alpha and beta double knock-out; ns, nonsignificant; SSC, side scatter. (TIFF) [file pbio.3000948.s005.tiff]

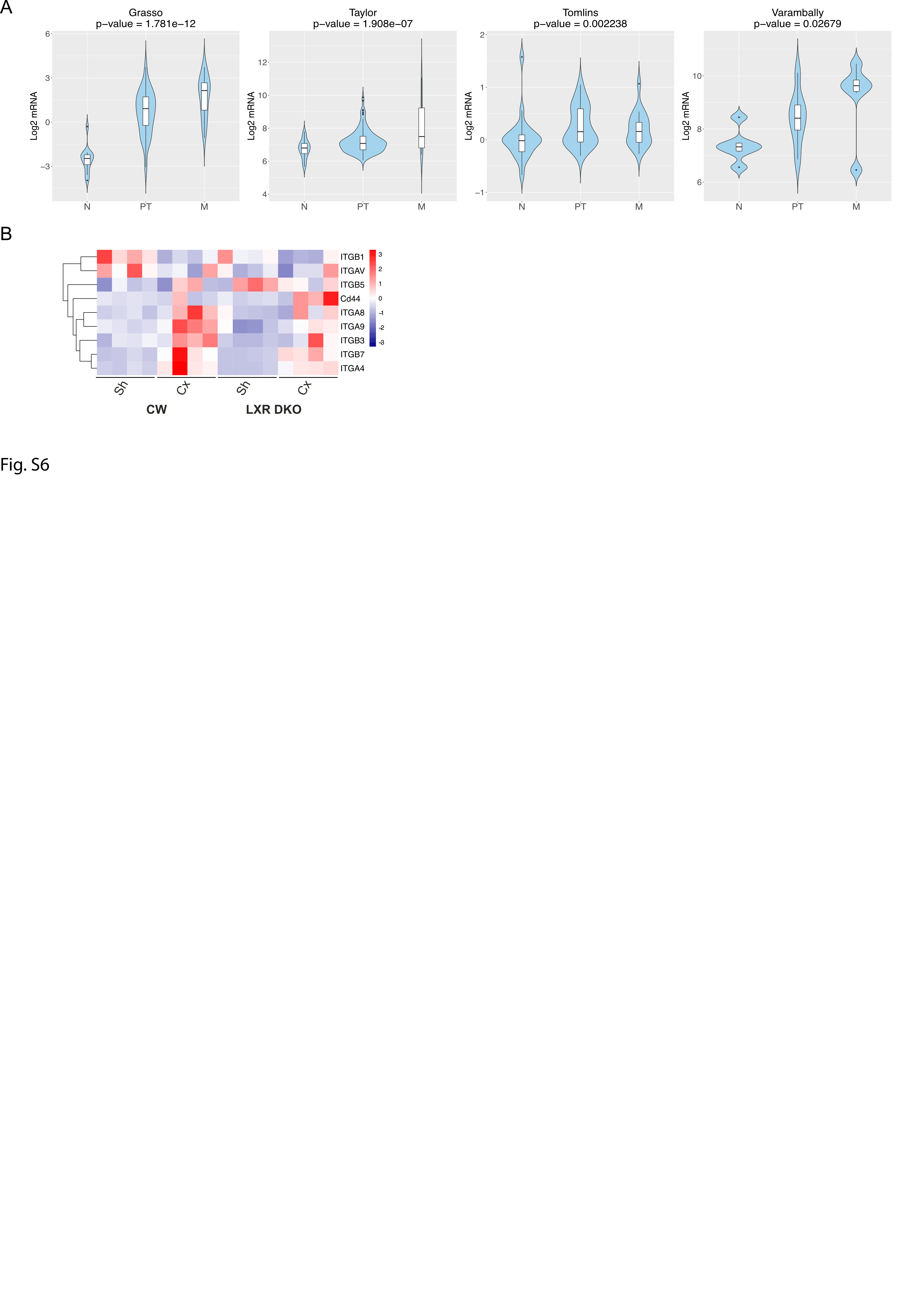

Supplement: S6 Fig — (A) OPN expression is associated with prostate cancer progression in publicly available prostate cancer patients’ cohorts [68–72]. (B) Heatmap of genes encoded various OPN receptors. CW, control wild-type; Cx, castrated; OPN, osteopontin; Sh, sham-operated. (TIFF) [file pbio.3000948.s006.tiff]

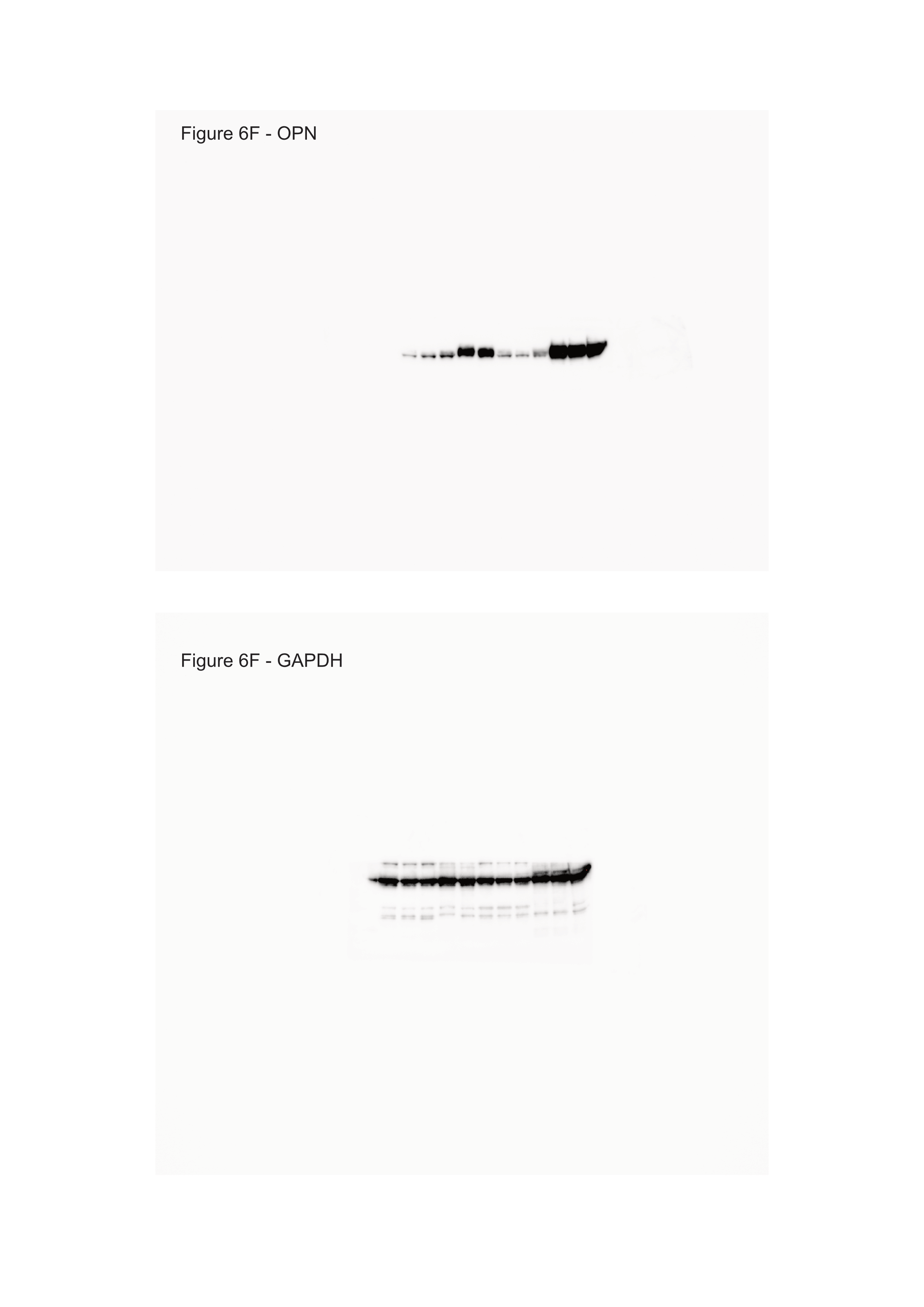

Supplement: S1 Blots — The file “S1_Blots” aggregates all uncropped and original western blots images. (TIFF) [file pbio.3000948.s007.tiff]
