# Supplementary material for: Absence of nuclear receptors LXRs impairs immune response to androgen deprivation and leads to prostate neoplasia
Source: PLoS Biol. 2020 Dec 7;18(12):e3000948. doi: 10.1371/journal.pbio.3000948 (PMC7752095; doi:10.1371/journal.pbio.3000948)
Supplement: S1 Table — (DOCX) [file pbio.3000948.s016.docx]

**S1 Table**

Primers used for RT-qPCR.

| Target | Forward Primer | Reverse Primer |
| --- | --- | --- |
| mm36b4 | GTCACTGTGCCAGCTCAGAA | TCAATGCTGCCTCTGGAGAT |
| mmAr | ATTGCCCATCTTGTCGTCTCC | ATCTTCTGGGATGGGTCCTCA |
| mmCcnb2 | GCCAAGAGCCATGTGACTATCCGG | CCTCCATCTGCACTGGTTTCACAGAG |
| mmCcne2 | GCATTCTGACCTGGAACCACAGATGA | GTCTCCCTATGAAGAGTGTATACTTCACAAAC |
| mmFkbp5 | CATCTTCACCAGGGCTTTGT | CTCAAACCCAAACGAAGGAG |
| mmIl6 | TGAAGGACTCTGGCTTTGTCT | ATGGATGCTACCAAACTGGAT |
| mmIl1b | GTTGATTCAAGGGGACATTA | AGCTTCAATGAAAGACCTCA |
| mmMki67 | AGTCTCTTGGCACTCACAGC | ATGGATGCTCTCTTCGCAGG |
| mmMme | TCTGTGGCCAGACTGATTCGTCA | AGCAGCATTGGGTCATTTCGGT |
| mmPbsn | AAGATAAATGAAGGCTCACCATTG | CATATTGATGTTTCAGGTTCCAGG |
| mmSpp1 | AGGTCCTCATCTGTGGCATC | TCTGATGAGACCGTCACT |
| mmTnf | CTGTAGCCCACGTCGTAGC | TTGAGATCCATGCCGTTG |
