# Supplementary material for: Absence of nuclear receptors LXRs impairs immune response to androgen deprivation and leads to prostate neoplasia
Source: PLoS Biol. 2020 Dec 7;18(12):e3000948. doi: 10.1371/journal.pbio.3000948 (PMC7752095; doi:10.1371/journal.pbio.3000948)
Supplement: S2 Table — (DOCX) [file pbio.3000948.s017.docx]

**S2 Table**

Antibodies and conditions used for immunohistochemical analyzes.

| Antibody | Clone | Reference | Supplier | Antigen Retrival Buffer | Dilution |
| --- | --- | --- | --- | --- | --- |
| AR | N-20 | sc-816 | Santa Cruz | Tris 10 mM EDTA 1 mM pH9 | 1/1000 |
| Cleaved Caspase 3 | ASP175 | 9661 | Cell Signaling | Citrate de sodium 10 mM,  Tween 0.05% pH 6 | 1/200 |
| CD45 | 30-F11 | 550539 | BD Pharmingen | without | 1/200 |
| CK8 | 1E8 | 904801 | Biolegend | Tris 10 mM EDTA 1 mM pH9 | 1/500 |
| F4/80 | A3-1 | MCA497GA | Bio-Rad | without | 1/500 |
| Ki67 | SP6 | M3060 | Spring Bioscience | Tris 10 mM EDTA 1 mM pH9 | 1/200 |
| OPN | AKm2A1 | sc-21742 | Santa Cruz | Vector | 1/1000 |
| pSTAT3 | D3A7 | 9145 | Cell Signaling | Tris 10 mM EDTA 1 mM pH9 | 1/50 |
