# Supplementary material for: Absence of nuclear receptors LXRs impairs immune response to androgen deprivation and leads to prostate neoplasia
Source: PLoS Biol. 2020 Dec 7;18(12):e3000948. doi: 10.1371/journal.pbio.3000948 (PMC7752095; doi:10.1371/journal.pbio.3000948)
Supplement: S3 Table — (DOCX) [file pbio.3000948.s018.docx]

**S3 Table**

Antibodies used for western blot.

| Antibody | Clone | Reference | Supplier | Molecular Weight (kDa) | Dilution |
| --- | --- | --- | --- | --- | --- |
| GAPDH |  | 9545 | Sigma Aldrich | 36 | 1/10000 |
| OPN | AKm2A1 | sc-21742 | Santa Cruz | 55 | 1/1000 |
