# Supplementary material for: Absence of nuclear receptors LXRs impairs immune response to androgen deprivation and leads to prostate neoplasia
Source: PLoS Biol. 2020 Dec 7;18(12):e3000948. doi: 10.1371/journal.pbio.3000948 (PMC7752095; doi:10.1371/journal.pbio.3000948)
Supplement: S4 Table — (DOCX) [file pbio.3000948.s019.docx]

**S4 Table**

Antibodies used for flow cytometry analysis.

| Antibody | Clone | Isotype | Reference | Supplier | Dilution |
| --- | --- | --- | --- | --- | --- |
| APC anti-mouse F4/80 | BM8 | Rat IgG2a, k | 123116 | Biolegend | 1/50 |
| APC anti-mouse CD4 | GK1.5 | Rat IgG2b, k | 100412 | Biolegend | 1/50 |
| PE anti-mouse CD19 | 6D5 | Rat IgG2a, k | 115508 | Biolegend | 1/100 |
| PerCP anti-mouse/human CD11b | M1/70 | Rat IgG2b, k | 101230 | Biolegend | 1/50 |
| PE anti-mouse CD11c | N418 | Armenian Hamster IgG | 117308 | Biolegend | 1/100 |
| Purified anti-mouse CD16/32 | 93 | Rat IgG2a, l | 101302 | Biolegend | 1/100 |
| Alexa Fluor 700 anti-mouse Ly-6C | HK1.4 | Rat 1gG2c, k | 128024 | Biolegend | 1/100 |
| Alexa Fluor 488 anti-mouse Ly-6G | 1A8 | Rat IgG2a, k | 127626 | Biolegend | 1/100 |
| PE-CF594 Rat Anti-Mouse CD45 | 30-F11 | Rat IgG2b, k | 562420 | BD | 1/100 |

| Isotype Control Antibody | Clone | Isotype | Reference | Fournisseur |
| --- | --- | --- | --- | --- |
| APC Rat IgG2a, κ | RTK2758 | Rat IgG2a, k | 400512 | Biolegend |
| APC Rat IgG2b, κ | RTK4530 | Rat IgG2b, k | 400612 | Biolegend |
| PE Rat IgG2a, κ | RTK2758 | Rat IgG2a, k | 400508 | Biolegend |
| PerCP Rat IgG2b, κ | RTK4530 | Rat IgG2b, k | 400630 | Biolegend |
| PE Armenian Hamster IgG | HTK888 | Armenian Hamster IgG | 400908 | Biolegend |
| Alexa Fluor 700 Rat IgG2c, κ | RTK4174 | Rat IgG2v, k | 400729 | Biolegend |
| Alexa Fluor 488 Rat IgG2a, κ | RTK2758 | Rat IgG2a, k | 400525 | Biolegend |
| PE-CF594 Rat IgG2b, κ | A95-1 | Rat IgG2b, k | 562308 | BD |
